# The cargo protein MAP17 (PDZK1IP1) regulates the immune microenvironment

**DOI:** 10.18632/oncotarget.21651

**Published:** 2017-10-06

**Authors:** José M. García-Heredia, Amancio Carnero

**Affiliations:** ^1^ Instituto de Biomedicina de Sevilla, IBIS/Hospital Universitario Virgen del Rocío/Universidad de Sevilla/Consejo Superior de Investigaciones Científicas, Seville, Spain; ^2^ Department of Vegetal Biochemistry and Molecular Biology, University of Seville, Seville, Spain; ^3^ CIBER de Cáncer, Instituto de Salud Carlos III, Madrid, Spain

**Keywords:** MAP17, oncogene, inflammation, cancer, inflammatory diseases

## Abstract

Inflammation is a complex defensive response activated after various harmful stimuli allowing the clearance of damaged cells and initiating healing and regenerative processes. Chronic, or pathological, inflammation is also one of the causes of neoplastic transformation and cancer development. MAP17 is a cargo protein that transports membrane proteins from the endoplasmic reticulum. Therefore, its overexpression may be linked to an excess of membrane proteins that may be recognized as an unwanted signal, triggering local inflammation. Therefore, we analyzed whether its overexpression is related to an inflammatory phenotype. In this work, we found a correlation between *MAP17* expression and inflammatory phenotype in tumors and in other inflammatory diseases such as Crohn's disease, Barrett's esophagus, COPD or psoriasis. *MAP17* expression correlated also with the markers of inflammation *HLAs, BBS10, HERC2, ADNP* and *PYCARD*. Furthermore, we found that MAP17 expression directly regulates NFAT2 and IL-6 activation, inducing the differentiation of monocytes to dendritic cells and suggesting a causal role of MAP17 in inflammation. Immunohistochemistry confirms local inflammation, mainly CD45^+^ cells, at the site of expression of MAP17, at least in tumors, Crohn's and psoriasis. Therefore, our data indicates that the overexpression of the protein MAP17 plays important role in diseases involving chronic inflammation.

## INTRODUCTION

Inflammation is a common defensive response that is activated after different harmful stimuli. It is a highly complex biological mechanism that allows the clearance of damaged cells and the removal of pathogens, initiating healing and regenerative processes [1, 2]. The inflammatory process activates signals that attract and activate inflammatory cells, such as macrophages, which generate and release cytokines and proinflammatory chemokines [3]. These molecules attract circulating leukocytes to the site of inflammation. After the elimination of harmful agents, this mechanism allows tissue repair to begin [4]. It is known that many cytokines activate regeneration-activating pathways such as those of YAP, Notch and Stat, which are also involved in stem cell activation [2, 5]. To end the inflammatory process, activated cells undergo apoptosis in a tightly regulated process that finishes after the phagocytosis of pathogens and cell debris [6]. However, when the inflammatory cells are incapable of eliminating the pathogen, inflammation may turn chronic, being characterized by a high level of leukocyte infiltration in damaged tissues. This chronic inflammatory condition contributed to the origin of many syndromes, including Crohn's disease, lupus, psoriasis and atherosclerosis. In addition, it has also been connected to Alzheimer's disease and cardiovascular disease [7, 8].

This chronic, or pathological, inflammation is also one of the causes of neoplastic transformation and cancer development [9]. Indeed, approximately 25% of tumors have an important association with chronic inflammation derived from an infection, especially stomach cancer [10]. Persistent gastritis caused by *Helicobacter pylori* increases the risk of developing gastric tumor by 75% [11]. In addition, infection with hepatitis B or C viruses increases the risk of hepatocellular carcinoma [12]. Further, chronic inflammation of the pancreas or prostate is commonly followed by the appearance of tumors in these organs [13]. Crohn's disease is another example, increasing the risk of developing colorectal cancer by up to ten-fold [14]. All these tumors, derived from a chronic inflammatory condition, are characterized by the presence of immune cells and mediators of inflammation. Indeed, leukocytes can form up to 50% of total tumor mass [15]. The interaction between cancer cells and macrophages stimulates these cells to produce proinflammatory cytokines such as IL-8, thereby attracting additional inflammatory cells [16, 17]. This inflammatory microenvironment has been accepted as an essential component of all tumors [1, 16]. Recent efforts have been dedicated to understanding tumor-elicited inflammation, the inflammatory reaction that allows tumor development and is detected in many solid malignancies.

MAP17 (DD96, PDZKIP1) is a small, nonglycosylated membrane-associated protein that is located on the plasma membrane and in the Golgi apparatus [18–20]. MAP17 has an N-terminal hydrophobic region of 13 amino acids, a double transmembrane region, and a C-terminal region of 61 amino acids [21]. The last 14 amino acids of the MAP17 C-terminal domain contain a PDZ-binding domain that allows the transport of this protein from the Golgi to the cell membrane. This domain allows its interaction with PDZK1 and its associated proteins, such as OCTN1, CFEX, URAT1 or NaPi-I/II [22, 23]. Although its physiological role in proximal tubules is not fully known, MAP17 contributes, as a cargo protein, to the membrane localization of many transporters in the cellular membrane and stimulates specific Na-dependent transport of glucose in *Xenopus* oocytes and human tumor cells [23, 24].

MAP17 works as an oncogene, increasing tumorigenicity when it is overexpressed [25–29]. Tumor cells overexpressing MAP17 have proliferative advantage over cells not expressing MAP17 [25]. Also, its overexpression in cancer cells reduced the percentage of apoptotic cells and induced increased growth ratios in mice tumors [25, 30]. Furthermore, the ectopic MAP17 expression induced an increment in stem cell-like properties, soft agar and tumorspheres assays, and increment in stem cell-like transcription [27, 30]. MAP17 downregulation by specific shRNA in cells that naturally express high levels of MAP17, reduced tumorigenic and cancer stem cell-like properties of cancer cells [27, 30, 31]. MAP17 overexpression activates Notch pathway due to direct interaction between MAP17 and NUMB in tumor cells [27]. This interaction is mediated by the 13 last amino acids of MAP17, causing an increment in nuclear NICD and increased expression of Notch target genes, like *HES1* or *HES5* [27]. In addition, MAP17 levels are correlated with tumoral progression, being higher in late-stage or metastatic tumors regarding benign tumors or normal tissues. MAP17 overexpression has been found in advanced stages in ovarian, cervical, laryngeal and prostate tumors [26]. Thus, a high percentage of advanced tumors, from 50 to 90%, exhibits high levels of MAP17, being this expression correlated with an increment in dedifferentiation [23, 30, 32].

In normal conditions, MAP17 is expressed only in kidney proximal tubule cells, whereas the rest of the organism does not express this protein and does not have to cope with its effects. Therefore, its overexpression in other cell types may be linked to an excess of membrane proteins that may be recognized as an unwanted signal, triggering local inflammation. We therefore decided to analyze whether its overexpression could be related to an inflammatory phenotype. Because MAP17 is highly expressed in a large number of tumors, we looked first for a correlation between MAP17 expression and inflammation in tumors and then in other diseases related to chronic inflammation. We found a clear correlation between MAP17 expression and these inflammatory diseases. Furthermore, we found that MAP17 is causal in the inflammatory phenotype because MAP17 expression regulates the expression of important inflammation-related genes.

## RESULTS

### *MAP17* expression correlates with inflammation in tumor samples

**Figure 1:**
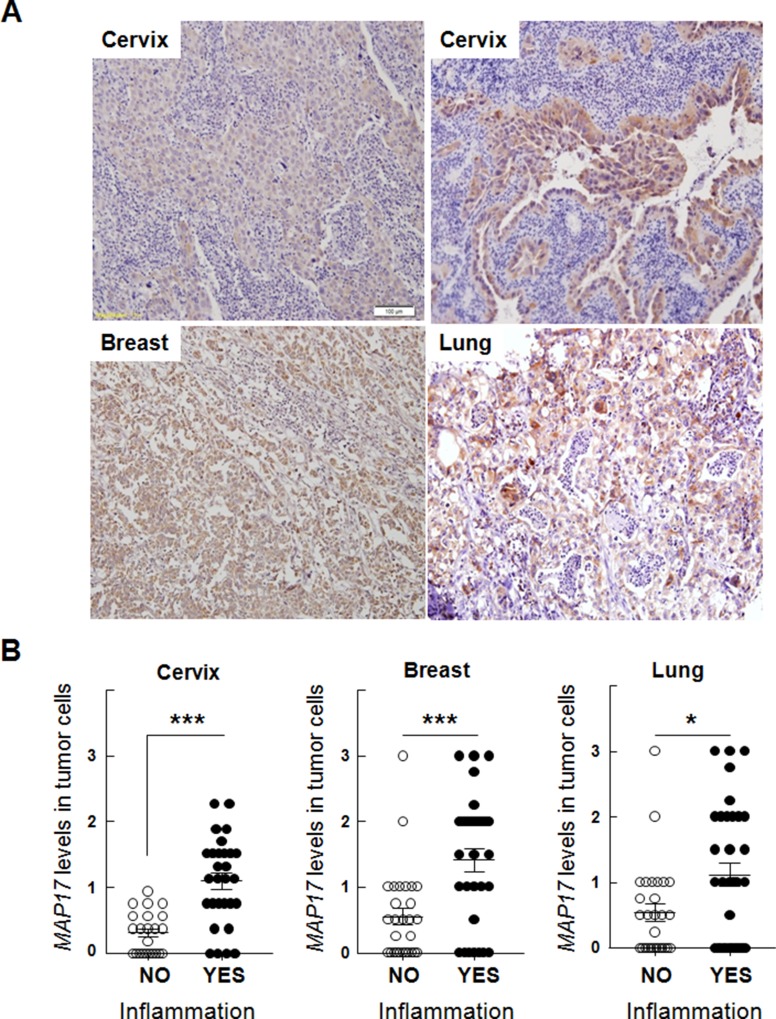
Correlation of MAP17 expression with inflammation in human tumors **(A)** Hematoxylin/eosin staining of histological slides of cervical, breast and lung cancer, showing a correlation of MAP17 levels with macrophage infiltration levels. **(B)** Quantification of the correlation of MAP17 expression with inflammation in human tumors. MAP17 expression was quantified in two independent sections of each sample, according to the intensity of the signal (0= no expression, to 3= very high expression), by double blind observation of two independent pathologists. Inflammatory infiltration was considered positive when the percentage of infliltrating cells was >5% of the total number of cells. Data include Student's T test for statistical analysis of the data. ^*^ = p < 0.05; ^**^ = p < 0.01; ^***^ = p < 0.001.

**Figure 2:**
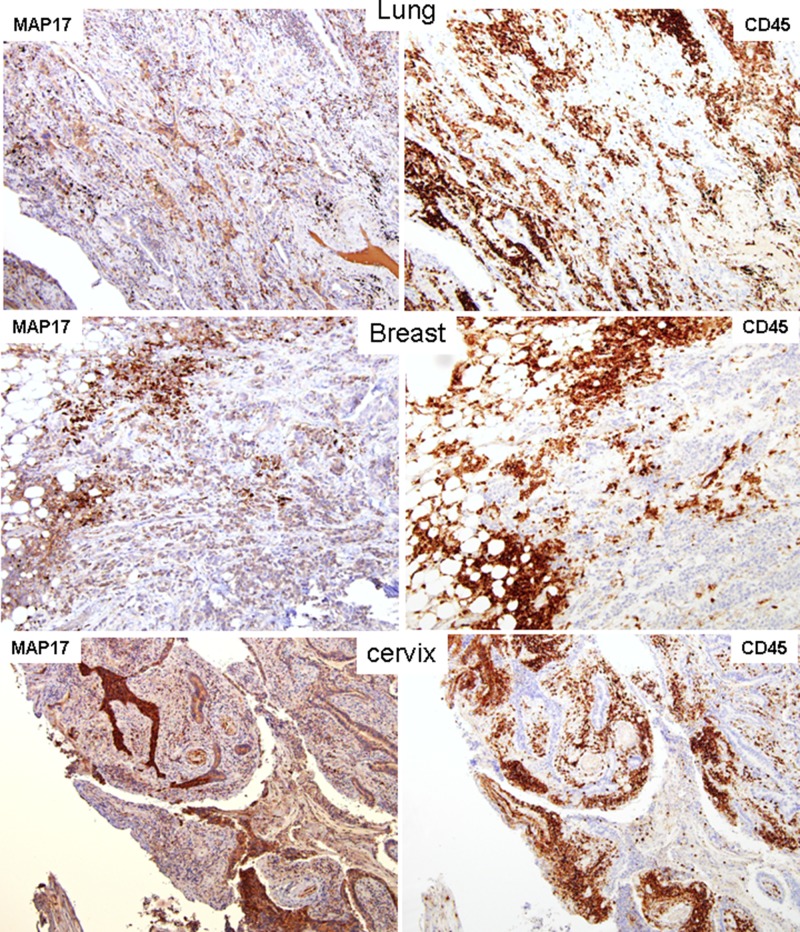
Correlation of MAP17 expression with inflammation (CD45) in human samples of tumors of different origin MAP17 and CD45 staining of serial sections of human samples of tumors of different origin. MAP17 staining in all samples showed high expression levels, correlated with the inflammatory marker CD45.

First, we analyzed a series of human cervix, breast and lung tumor samples [23, 33, 34], finding that *MAP17*-positive tumors usually correlated with higher levels of inflammatory infiltration (Figure 1A). Although the quantity of inflammatory cells infiltrating the tumor varied among the different tumor samples, it was clearly detected that, in these tumor types, *MAP17* is significantly associated with inflammation (p < 0.05) (Figure 1B). These cells are mostly CD45+ (Figure 2) and its expression strongly correlated with MAP17 expression. To explore the different composition of these inflammatory populations we analyzed the CD4^+^ (T cells “helper”) and CD8^+^ (T cells “cytotoxic”) percentages, since this is relevant for efficacy of some antitumor therapies and have predictive value [35]. We have performed this study in breast, cervix, and lung tumor samples. We have observed that, in general, CD4 and CD8 positive T cells can be observed infiltrating tumors (Supplementary Figures 1-3). Interestingly, in most of the samples, the percentage ratio of CD4^+^/CD8^+^ cells is >1. However, we have not found a correlation of the percentages of CD4 nor CD8 T cells with those of MAP17 expression (Supplementary Table 1).

**Figure 3:**
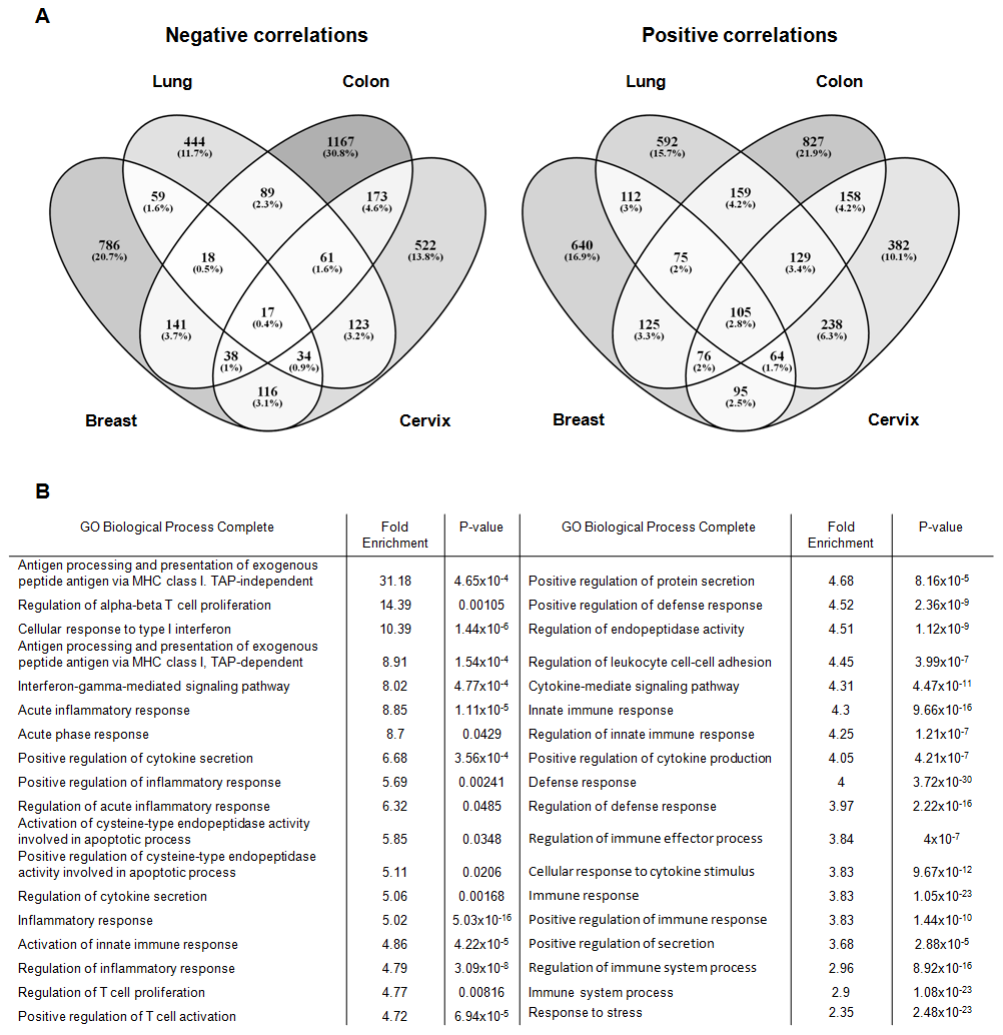
**(A)** Venn diagrams of the significant genes found negatively and positively correlated with *MAP17* in lung, breast, colon and cervical tumors, showing that, for negative correlations, 17 genes appears equally negatively correlated and 105 genes positively correlated with MAP17 in the four tumor types considered (p-value < 0. 05). **(B)** GO Biological terms related to inflammation that appear with genes positively correlated with *MAP17* in at least three of the four types of tumors.

To more deeply explore the correlation between MAP17 and inflammation, we next looked for genes that correlated with *MAP17* in 4 tumor types: lung, cervical, breast and colorectal tumors (Supplementary Figure 4). To this end, we selected public datasets from transcriptomic analysis of these 4 tumor types (Supplementary Table 2). We first obtained the genes that correlated with *MAP17* in all tumor types and then compared the individual tumor types to obtain a map of common genes correlating to *MAP17* expression in various tumors. Thus, based on the bioinformatics analysis, we obtained four lists of genes negatively correlated with *MAP17* (845, 1209, 1704, and 1084 genes for lung, breast, colon, and cervical tumors, respectively) and another four lists of genes positively correlated with *MAP17* (1473, 1292, 1653, and 1246 genes for lung, breast, colon, and cervical tumors, respectively). To find genes common to different tumors, we compared the lists of genes, identifying a substantial number of genes that appear in at least two of the selected tumors (Figure 3A). Through these comparisons, we obtained 105 genes positively correlated with *MAP17* and 17 genes negatively correlated with *MAP17* in all tumor types considered (Supplementary Table 3). If we consider the presence of genes in at least 3 of the 4 different tumors, the number increases to 168 genes negatively and 449 genes positively correlated with *MAP17* (Supplementary Tables 4 and 5).

### Gene ontology analysis of genes correlating with *MAP17*

To detect pathways that are significantly altered, we analyzed genes positively correlated with *MAP17* in at least 3 of the 4 tumors considered (Supplementary Table 5). Using enrichment analysis, we found that some of the altered pathways identified using the PANTHER database were correlated with membrane signaling and inflammation (for example, antigen processing and presentation of exogenous peptide antigen via MHC class I, TAP-independent, fold enrichment: 31.18; acute inflammatory response, fold enrichment: 8.85; positive regulation of protein secretion, fold enrichment: 4.68) (Figure 3B). In addition, we detected numerous proteins that are secreted or that can be found attached to the extracellular side of the cell membrane (Supplementary Table 6). In addition, most of the GO terms found were related to a secretory phenotype (Figure 3B). Three of the lowest p-values corresponded to defense response (p-value: 3.72×10^-30^), response to stress (2.48×10^-23^) and immune response (1.05×10^-23^). Further, inflammatory response is also one of the GO terms with a relatively low p-value (5.03×10^-16^). In addition, we found overrepresentation of the human leukocyte antigen (HLA) family (see Supplementary Table 3), with nine of its members (F, G, B, J, C, E, DMA, A and DRA) positively correlated with *MAP17* in at least 15 of the total databases considered, as well as another 6 (DOB, DPA1, DMB, DQB1, DMB and DQB1) appearing in at least 10 of the analyzed databases (see Supplementary Figure 5). Genes of this family have been related to different inflammatory phenotypes, mainly due to changes in its expression levels [36–39].

*PYCARD, CASP1* and *CASP8*, three of the genes positively correlated with *MAP17*, have been typically connected to inflammation as essential members of the inflammasome platform that triggers inflammatory response [40], appearing highly represented in the four types of tumors considered (see Supplementary Table 3). Other components, such as *CASP5*, appeared in three of the selected tumors, while *NLRP1, NLRP3* or *NLRC4*, also components of inflammasome, appeared in one or two of the selected databases. Interleukin-1β (IL-1B), a proinflammatory cytokine that is produced as an inactive cytoplasmic precursor and is cut by caspase-1 (*CASP1*) [40], also appeared in all tumors. Not only does *IL-1B* appear positively correlated with *MAP17* in all tumors, other interleukins including *IL-15, IL-18, IL-1A, IL-32* and *IL-7* and interleukin receptors including *IL-10RB, IL-17RC* and *IL-2RG* appear in at least three of the tumor types (Supplementary Table 5). All these elements have been correlated with an increase in inflammation [41, 42].

Regarding negative correlations, we found no prominent pathways connected to *MAP17*, although it is noteworthy that one of the GO terms was “developmental process”, suggesting an inhibition of cell differentiation, a feature of cancer cells (see Supplementary Table 7). The analysis of common genes allowed us to find another GO term related to differentiation, “Anatomical structure development”. In addition, we found that “chromosome organization” was negatively correlated with *MAP17*, and this process may be related to the chromosome instability of cancer cells.

**Figure 4:**
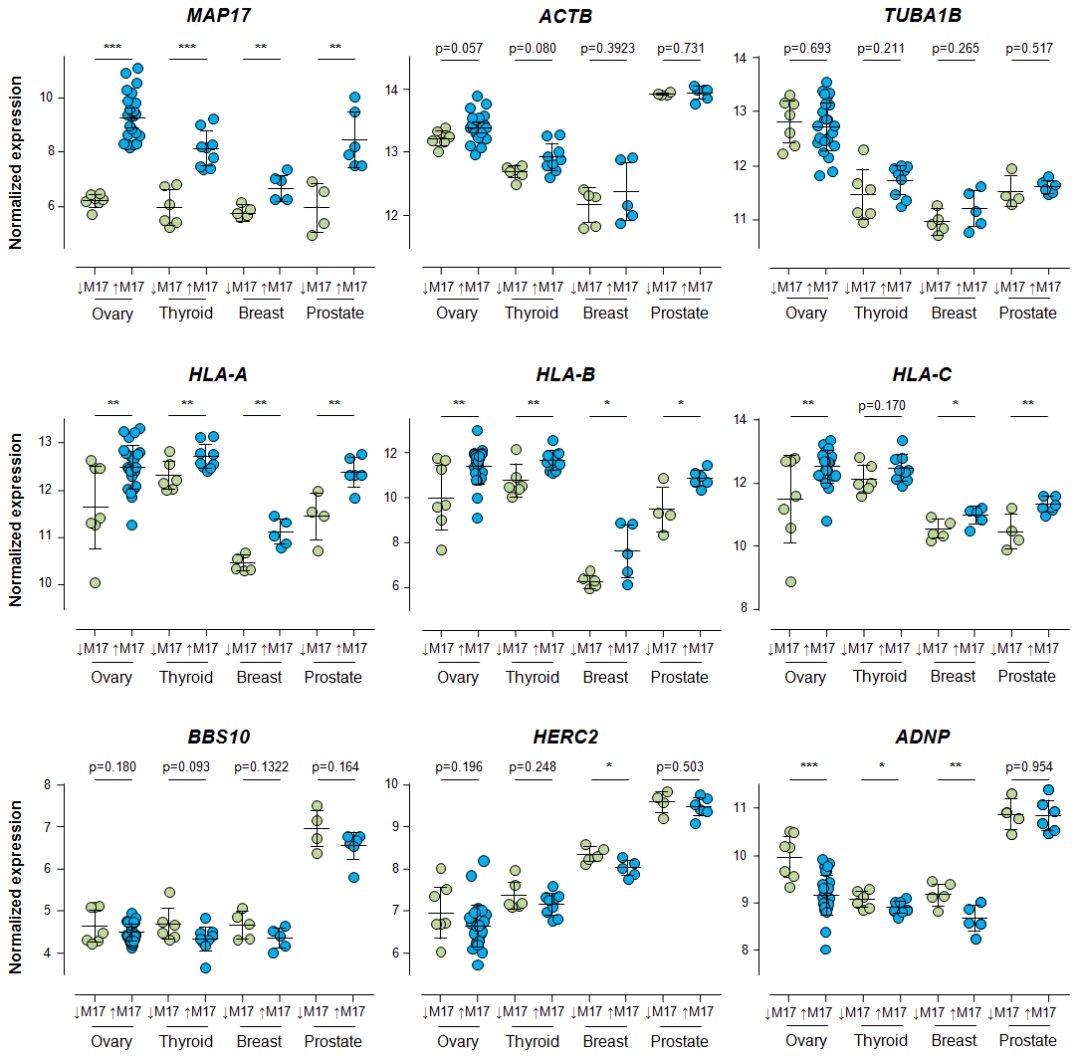
Expression of HLA-A, HLA-B, HLA-C, BBS10, HERC2 and ADNP in breast, thyroid and ovarian cancer samples, grouped by differential expression levels of MAP17, obtained through RNA normalization of public transcriptomic arrays, as described in Methods, Bioinformatic analysis section Once grouped, Student's T test for statistical analysis of the data was applied. ^*^ = p < 0.05; ^**^ = p < 0.01; ^***^ = p < 0.001. All significant differences found in ovary, thyroid and prostate tumors showed the same correlation with MAP17 that the previously found for breast, colon, cervix and lung tumors. In the case of non-significant differences, the tendency of the samples points, also, the same direction.

Inflammatory response is commonly positively correlated with *MAP17* in tumoral samples. To determine whether our results were a common feature in other tumors (ovary, thyroid and prostate, maintaining breast as a positive control), connecting MAP17 with a higher inflammation phenotype, we selected 3 genes for each type of correlation with *MAP17*: *HLA-A, HLA-B* and *HLA-C* for positive correlations and *BBS10, HERC2* and *ADNP* for negative correlations. HLAs positive correlation to inflammation have been largely described [43]. BBS10 (Bardet-Biedl Syndrome 10) missense mutations have been correlated with higher inflammatory markers [44]. ADNP (Activity-Dependent NeuroProtector) expression has been connected with a reduction on the expression of pro-inflammatory cytokines [45]. HERC2 (HECT and RLD domain containing protein 2 ortholog) is a probable E3 ubiquitin-protein ligase. Comparative genetic analysis of inflammatory bowel disease and type 1 diabetes implicates HERC2 loci [46]. *HERC2* has been genetically associated with Crohn's disease [47], sarcoidosis and its acute and chronic subphenotypes [48]. We analyzed the behavior of all these genes in different tumor samples and compared them to that of *MAP17*. We identified significant increases in the expression of *HLA-A, HLA-B* and *HLA-C* in the four tumor types considered, whereas *BBS10, HERC2* and *ADNP* showed a clear tendency toward decreased expression when *MAP17* is overexpressed (Figure 4). The two controls, *ACTB* (actin) and *TUBA1B* (tubulin), did not show significant differences in any of the databases selected (Figure 4), confirming the specificity of our data. *PYCARD* and *CASP1*, two of the elements of the inflammasome platform implicated in the IL-1B maturation process, appear also significantly increased, correlating with high levels of *MAP17* (Supplementary Figure 6). These results showed that *MAP17* overexpression can be connected with an inflammatory phenotype, and we therefore decided to analyze whether this overexpression acts as a marker of inflammation or may be an indirect cause of it.

### MAP17 expression regulates inflammatory genes

**Figure 5:**
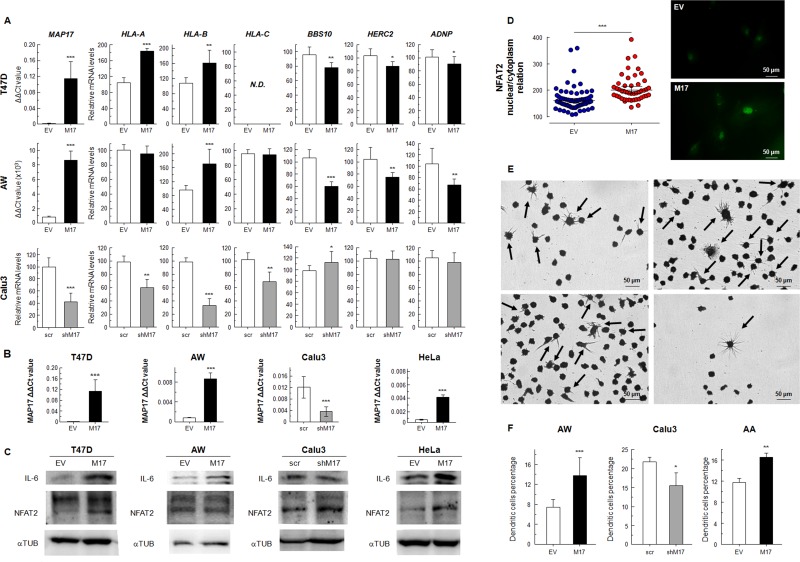
**(A)** mRNAexpression levels of *HLA-A, HLA-B, HLA-C, BBS10, HERC2* and *ADNP* in cancer cells transfected for overexpression of *MAP17* (T47D, AW) or knockdown of *MAP17* with specific shRNA (Calu3). **(B)** *MAP17* expression in human cells transfected to induce its overexpression (T47D, AW, HeLa) or knockdown (Calu3). **(C)** WB of NFAT2 and IL-6, pro-inflammatory proteins, in cancer cells that overexpress *MAP17* (T47D, AW, HeLa) or with its expression downregulated (Calu3). IL-6 is clearly overexpressed in cell lines with higher MAP17 levels, while NFAT2 appears also with higher levels in the cells that overexpress MAP17. **(D)** NFAT2 nuclear proportion is increased due to MAP17 overexpression. Left) nuclear/cytoplasm relation in AW cells transfected with EV or MAP17 overexpressing vector, according to the fluorescence microscopy images obtained of NFAT2 expression. Right) Fluorescence microscopy of AW EV or AW MAP17 cells. The latter exhibits higher nuclear levels of NFAT2 **(E)** U937 cells attached to the plate, after being exposed to conditioned cell media (See Methods). Some cells showed the typical form of differentiated dendritic cells (black arrows). **(F)** Percentage of attached U937 cells for different conditioned media. The percentage of cells with dendritic morphology is higher for cells with higher MAP17 levels. All experiments were repeated a minimum of three independent times in triplicate. All figures include Student's T test for statistical analysis of the data. ^*^ = p < 0.05; ^**^ = p < 0.01; ^***^ = p < 0.001.

First, we generated cells in which MAP17 was overexpressed, such as in breast tumor cells T47D, or in the sarcoma cells AW [49]. Previously, we showed that MAP17 overexpression in these cells induces an increment in tumorigenic properties [50, 51]. Here, we detected that MAP17 overexpression caused an increased expression of *HLA* genes, while significantly decreasing (p < 0.05) the expression of *BBS10, HERC2* and *ADNP* (Figure 5A). Furthermore, using specifically targeted shRNA, we downregulated *MAP17* in Calu3 lung tumor cells, which endogenously express high levels of *MAP17* (Figure 5A). In these cells, *MAP17* downregulation causes a clear reduction in the expression of *HLAs* while increasing *BBS10* expression. Although no increase in *HERC2* or *ADNP* was observed in Calu3 cells, a decrease in *PYCARD* was detected when MAP17 was downregulated (Supplementary Figure 7). Therefore, we concluded that *MAP17* expression regulates the expression of inflammation-related genes, suggesting a causal effect of *MAP17* in this phenotype (Figure 5A).

In order to determine the mechanism induced by MAP17 overexpression in tumor cells, we looked for NFAT2 and IL-6 expression, known attractant of inflammasome. NFAT2 belongs to a family of transcription factors that modulate the inflammatory response [52], while IL-6 has been connected to chronic inflammation, being produced at the site of inflammatory response [53]. In all the tumor cells overexpressing MAP17 (Figure 5B), we detected a clear increment in the expression of IL-6, while NFAT2 was clearly increased in HeLa cells with small changes in the other considered cell models, showing that MAP17 has a real role as an inflammatory inducer (Figure 5C). However, this increment might not be related with an increment in gene transcription, so we analyzed NFAT2 expression in AW cells, finding a higher nuclear/cytoplasm NFAT2 ratio in cells overexpressing MAP17 (Figure 5D).

Finally we tested whether the overexpression of MAP17 liberates proinflammatory inducers to the media and the ability of this media to induce the differentiation of monocytes to macrophages. To this end we cultured the U937 monocyte cell line in conditioned media (1:1) from AW and AA cells overexpressing *MAP17*, in addition to Calu3 cells with downregulated *MAP17* expression. Although we tested U937 with T47D conditioned media, the number of derived attached cells was so low 72 hours after the beginning of the experiment that we could not find any significant differences, so we used AA cells, a previously characterized cell line where MAP17 overexpression also increased tumorigenic properties [50]. We expect this experiment reconstitutes *in vitro* the physiological conditions found at MAP17-positive tumor site. We found that part of the U937 monocyte cell line attached to the plate, showing cells exposed to MAP17-conditioned media a significantly higher percentage of dendritic cells (Figure 5E, 5F), confirming our hypothesis of the relevant causal role of MAP17 in inflammation. In fact, IL-6 has been previously connected to monocyte differentiation to dendritic cells [54].

### *MAP17* overexpression is a common feature in chronic inflammatory diseases

**Figure 6:**
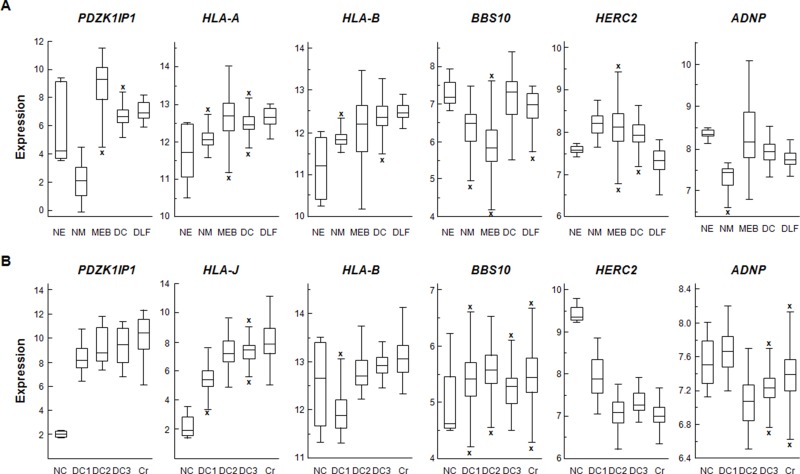
**(A)** Normalized expression levels of *MAP17* (*PDZK1IP1*), *HLA-A, HLA-B, HLA-C, BBS10, HERC2* and *ADNP* in inflammatory diseases. NE: Normal Epithelium (Shelhamer database), NM: Normal Macrophage (Salazar database), MEB: Mixed Barrett's Esophagus, DC: COPD (Tiley database), DLF: Lung Fibrosis (Meltzer database). **(B)** Normalized expression levels of *MAP17, HLA-B, BBS10, HERC2* and *ADNP* in colon. NC: Normal Colon (Vivier database), DC1: Diseased colon (Sleiman database), DC2: Diseased colon (Salas database), DC3: Diseased colon (Seidelin database), Cr: Disease Crohn's disease (Arijs database). In both cases, the differences in the expression of the considered genes between normal and inflammatory samples are usually in the same direction that the previously found in tumoral samples. Data were obtained through MegaSampler, included in R2 server and analyzed using the MAS5.0 algorithm and are presented as the log2 transform.

After analyzing the connection between MAP17 and the inflammatory profile in tumoral samples, we wondered whether this profile was limited to cancer or could be considered a general effect. Therefore, we analyzed this gene profile (*MAP17, HLA-A, HLA-B, HLA-C, BBS10, HERC2* and *ADNP*) in non-tumoral samples of inflammatory syndromes, using chronic obstructive pulmonary disease (COPD), lung fibrosis and Barrett's esophagus to represent the inflammatory state and using both normal macrophages and epithelia to determine basal levels. We observed a similar pattern, with a high expression level of *MAP17* in inflammatory diseases compared to normal epithelial cells, connected to increased expression of *HLA-B* and *HLA-J*, members of the HLA family found in our screening, and downregulation of *BBS10, HERC2* and *ADNP* (Figure 6A). In addition, we also evaluated Crohn's disease and three colon inflammation databases, using normal colon as a control. Again, we found increased expression of *HLA* genes, correlating with increased *MAP17*, while *BBS10, HERC2* and *ADNP* generally showed reduced expression (Figure 6B).

**Figure 7:**
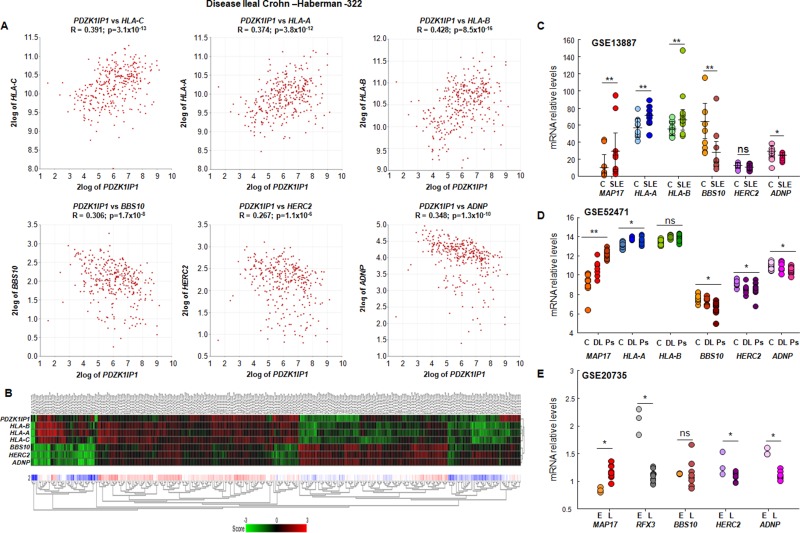
Correlation of MAP17 with proinflammatory molecules in ileal Crohn's disease **(A)** Correlation of *MAP17* (*PDZK1IP1*) with *HLA-A, HLA-B, HLA-C, BBS10, HERC2* and *ADNP* in ileal Crohn's disease, showing that MAP17 is positively correlated with *HLA-C, HLA-A* and *HLA-B*, and negatively correlated with *BBS10, HERC2* and *ADNP*. **(B)** Heatmap of *MAP17* (*PDZK1IP1*) with *HLA-A, HLA-B, HLA-C, BBS10, HERC2* and *ADNP* in ileal Crohn's disease, showing that, in general, *MAP17* is associated with low levels of *BBS10, HERC2* and *ADNP* and with high levels of *HLAs*. **(C, D** and **E)** Expression levels of *MAP17, HLA-A, HLA-B, HLA-C, BBS10, HERC2* and *ADNP* in lupus and psoriasis. **(C)** C: control (n=10); SLE: systemic lupus erythematosus (n=10); **(D)** C: control (n=13), DL: discoid lupus (n=7); Ps: psoriasis (n=18); **(E)** E: 6-8 week (n=3) TC RB^+^/CD4^++^/B220^-^ cells; L: 16-24 week (n=9) TC Rb^+^/CD4^+^/B220^-^/CD44^+^ cells. Disease progression showed a progresive increment in MAP17 expression levels. Data include Student's T test for statistical analysis of the data. ^*^ = p < 0.05; ^**^ = p < 0.01; ^***^ = p < 0.001.

To increase the accuracy of our data, we selected a large database of ileal Crohn's disease (GSE57945), a chronic inflammatory disease with no known origin, to analyze *MAP17* expression and its correlation to the six selected inflammatory genes. We found positive correlations between *MAP17* and the three *HLA* genes and negative correlations between *MAP17* and *BBS10, HERC2* and *ADNP* (Figure 7A). Indeed, a heatmap of the individual datasets using *MAP17* and the selected six genes as classifiers clearly showed the correlation with the six inflammatory genes (Figure 7B).

Lupus and psoriasis are another two inflammatory-related diseases, in this case affecting skin although of very different inflammatory physiology. To analyze the possible connection between *MAP17* and these diseases, we selected three databases corresponding to systemic lupus erythematosus (GSE13887), a mixed database with discoid lupus erythematosus and psoriasis (GSE52471), and a database for a lupus model in mice (GSE20735). In all of them, we observed a clear increase in *MAP17* expression (Figure 7C, 7D, 7E). Considering both the first database and lupus patients from the second database (SLE from Figure 7C and DL from Figure 7D), we observed significant differences in almost all of our selected genes when we separated individuals into two groups: low and high *MAP17* expression. In addition, in a mouse model that simulates lupus progression, animals experienced an increased expression of *MAP17* connected with a decrease in *RFX3*, a negative regulator of *HLAs*, and decreases in *HERC2* and *ADNP* (Figure 7E). Finally, a similar profile was found in psoriasis, with an increase in *MAP17* correlating to increased expression of HLAs and decreased expression of *BBS10, HERC2* and *ADNP* (Ps from Figure 7D).

**Figure 8:**
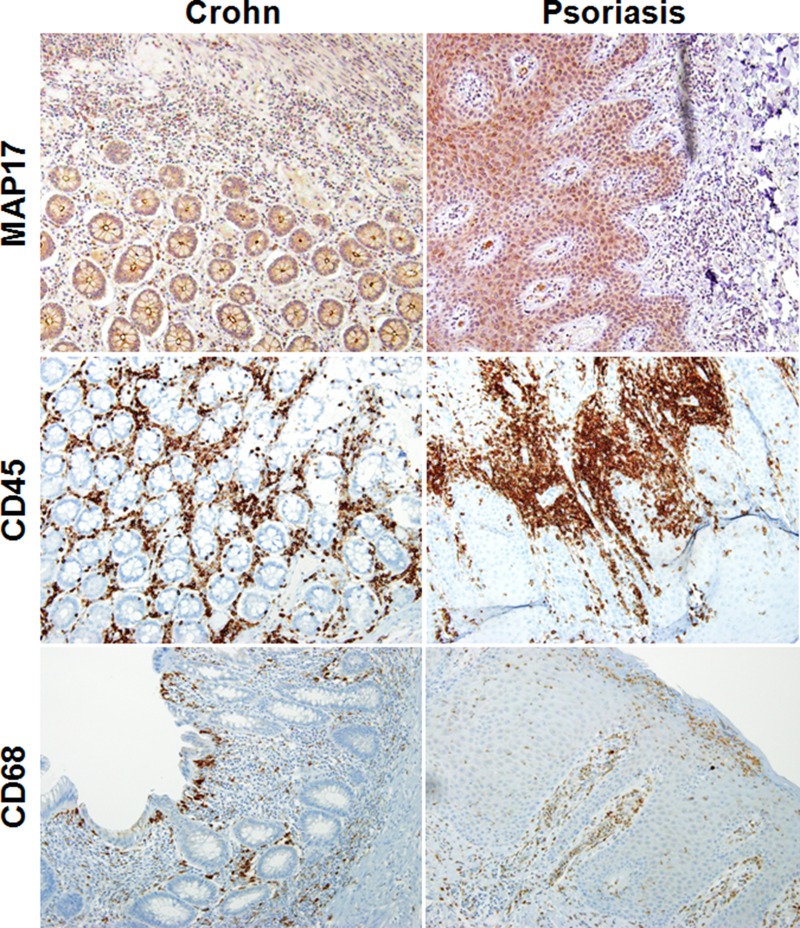
Correlation of MAP17 expression with inflammation in human samples of Crohn's disease and psoriasis MAP17, CD45 and CD68 staining of histological slides of human samples of psoriasis and Crohn's disease. MAP17 staining in both inflammatory diseases showed high expression levels, correlated with the inflammatory markers CD45 and CD68.

To finally confirm these data, we selected 20 samples of human Crohn's disease and 20 samples of human psoriasis and tested the expression of MAP17 in these tissues. We found that 20 out of 20 psoriasis samples showed a clear presence of MAP17 in the epithelial parenchyma. These MAP17-positive cells appear surrounded by inflammatory cells (Figure 8). Similar data were obtained for samples from human Crohn's disease; 75% of the samples analyzed showed clear staining for MAP17 in epithelial cells that were surrounded by inflammation (Figure 8). In all positive cases, we could observe a clear plasma membrane localization of MAP17. We also could observe a clear staining for CD45 and CD68 markers, two previously described specific surface markers of inflammation (Figure 8 and Supplementary Figures 8, 9 and 10). CD45, known also as common leukocyte antigen or receptor-type tyrosine-protein phosphatase C, is a very abundant leukocyte cell surface, being its expression restricted to haematopoietic cells [55]. CD68, a selective marker for monocytes and macrophages, have been previously connected to inflammatory diseases, like inflammatory bowel disease or Crohn's disease [56, 57].

Thus, our data confirm that the expression of the cargo protein MAP17 is a determinant of the induction of chronic inflammation and may be involved in the initiation of illnesses such as Crohn's disease, psoriasis, COPD and cancer.

## DISCUSSION

MAP17 has been found to be overexpressed in more than 50% of all tumors, also showing increased expression during tumor progression [26, 58, 59]. This protein, through its PDZ-binding domain, acts as a protein carrier from the Golgi apparatus to the cell membrane. However, according to our results, MAP17 not only triggers the attraction of inflammatory cells by increasing protein membrane loading but also modifies the expression of genes connected to inflammation, showing a clear induction of the inflammatory profile. First, MAP17 expression correlated with chronic inflammatory diseases. Second, within the list of genes related to *MAP17* expression, there are numerous genes involved in the inflammasome; therefore, inflammatory pathway-related genes positively correlated with *MAP17*. Finally, *MAP17* expression appeared to regulate the expression of inflammation-related genes, through induction of genes like NFAT2 and IL-6. Therefore, we suggest that the expression of MAP17 triggers chronic inflammation in various inflammatory diseases such as Crohn's disease, Barret's esophagus, lupus, psoriasis and COPD. Because MAP17 appears highly correlated with the infiltration of inflammatory cells in cancer, we can also suggest that MAP17 expression triggers chronic inflammation in many tumors. However, MAP17 is clearly not the only determinant of chronic inflammation because some samples of tumors, lupus, COPD and other inflammatory diseases showed no expression of MAP17.

We have shown that, in tumors, MAP17 activates components related to the inflammatory phenotype such as HLA molecules, proteins belonging to the major histocompatibility complex (MHC) family that can be induced in tumor cells as a result of local inflammation. HLA proteins are involved in antigen presentation to lymphocytes through a mechanism that involves proteasomal degradation of the antigen, its transport to the endoplasmic reticulum and its externalization to the plasma membrane [60, 61]. It has been reported that tumoral cells can upregulate HLA-A, HLA-B and HLA-C expression, decreasing the level of macrophage activation [62, 63]. In addition, it has been reported that the presence of these proteins in the plasma membrane inhibits the cytolytic function of natural killer cells [64, 65]. This inhibition of the activation of macrophages and natural killer cells occurs through the interaction of HLA proteins with the receptors LILR (Leukocyte immunoglobulin-like receptor) and KIR (Killer cell immunoglobulin-like receptor), negatively regulating their function [62]. MAP17, which is overexpressed in almost 50% of advanced carcinomas, increases its expression with the malignance of the tumor [32]. It has been previously shown that MAP17 is overexpressed in tumor cells due to progressive demethylation of its promoter [26, 66]. As a consequence of increased MAP17 levels, cell tumorigenic properties are increased, due to Notch pathway activation through NUMB abduction [50]. It has been shown that, through Notch pathway activation, NFAT pathway and IL-6 transcription are activated [67, 68]. In fact, we found *NFAT2* positively correlated with *MAP17* in five of our considered datasets (data not shown), and NFAT2 protein levels are significantly higher in cells overexpressing MAP17, being preferentially translocated to nuclei. Correlating with what has been previously shown [69], IL-6 levels are also increased by MAP17 expression. Addition of two proteins well connected to inflammatory response, IL-6 and Interferon-γ (IFNG), to cells induced expression of both HLA class I and class II [70–72]. In fact, IFNG receptors *IFNGR1* and *IFNGR2* appear overrepresented as genes positively correlated with *MAP17* (Supplementary Tables 3 and 5). Here, we have shown that MAP17 directly regulates HLA expression in tumor cells, and we have correlated MAP17 overexpression in different tumors with higher levels of these MHC genes. In addition, we found that, when MAP17 is overexpressed in tumoral tissues, there is an increase in macrophage infiltration, a clear marker of inflammation. Now, we can hypothesize that these macrophages should be inhibited due to the HLA expression guided by MAP17.

On the other hand, the large inflammatory response is a common feature of many tumors. This inflammatory response may not be the origin but act synergistically during the process of tumorigenesis. It is possible that the expression of the MAP17 proteins acts as an immune chemoattractant, and this immune presence then collaborates with neoplastic alterations. Enhanced inflammation surrounding target tissues could be a tumor promotion mechanism led by MAP17 deregulation controlling the development of hematopoietic cells that regulate inflammation, and mediate the responses of target cells to inflammatory cytokines. Mechanistically, high MAP17 levels increase the recruitment of tumor/inflammation associated macrophages, MDSCs, mast cells, and neutrophils to the target tissue, by increasing IL-6 locally and probably other cytokines or chemokines. It is possible that the increase in these cytokines in the extracellular media surrounding tumor cells might promote tumorigenesis by activating the NFkB and/or STAT3 pathways [73].

Therefore, it is possible that MAP17-positive tumors may be a target for immunotherapy. New antitumor therapy focused on inhibiting these inhibitory signals may be useful for targeting these MAP17-positive tumors. However, this is a preliminary hypothesis that must be experimentally tested.

Although we have described that MAP17 is commonly overexpressed in advanced tumors, its overexpression induces a higher level of tumorigenesis and increases ROS. The analysis of the correlation of MAP17 expression levels with survival in different tumors is complex. MAP17 levels can be considered as a marker of good prognosis in some tumors as cervix, treated with cisplatin and radiotherapy, strongly inducers of ROS [23]. However, in other tumors such as sarcoma treated with doxorubicin, MAP17 is a marker of worse prognosis [29]. Our group has also shown that cells with high levels of MAP17 are more sensitive to bortezomib treatment due to higher ROS production [74]. In addition, multiple myeloma patients, treated with bortezomib, showed higher levels of MAP17 associated to better prognosis [74].

In the present work we also found MAP17-positive tumors surrounded by CD4^+^ and CD8^+^ T cells in the tumor microenvironment. Since these cells are also involved in the overall response to treatments, the relative levels of these cells have been reported as good predictors of the patient survival in different tumors [35, 75]. Therefore, the survival observed in MAP17-positive patients may be a complex factor depending on the production of ROS and also related to the CD4/CD8 ratio of cells in the tumor microenvironment.

Nonetheless, the presence of immune cells in tumors constitutes positive feedback for oxidative stress, stimulating DNA damage and subsequent genome instability [1, 76]. However, leukocytes and their failed cell clearance must not be seen as a cause of tumor progression. Indeed, the appearance of mutations in damaged cells can also activate the immune response. Altogether, the activation of the inflammatory response by genetic alterations induces the appearance of a higher number of mutations [77]. However, it must be noted that not all chronic inflammatory diseases increase the risk of developing tumors. Indeed, some such diseases, like psoriasis, can reduce the probability of cancer [78]. In this regard, it is interesting to remark that when MAP17 is overexpressed in naïve cells, these undergo senescence [33], therefore affecting the ration of tumor initiation. How MAP17 expression may affect different tissues needs to be thoroughly studied.

In addition to the expression profile of MAP17 in tumoral samples, we also analyzed the same profile of MAP17-related genes in up to seven inflammatory diseases (COPD, lung fibrosis, Barrett's esophagus, Crohn's disease, colon inflammation, lupus and psoriasis). Most of the genes that we found correlated with MAP17 (both positively and negatively) showed the same pattern in all of these diseases. We have recently shown that MAP17 overexpression rendered tumor cells more susceptible to treatment with bortezomib, a proteasome inhibitor [29, 74]. In fact, it has been reported that bortezomib decreases the inflammatory response [79–81]. Thus, MAP17 may be useful as a biomarker of susceptibility to proteasome inhibitors for the treatment of chronic inflammatory diseases.

## MATERIALS AND METHODS

All methods were performed in accordance with the relevant guidelines and regulations of the Institute for Biomedical Research of Seville (IBIS) and University Hospital Virgen del Rocio (HUVR). All conform to the provisions of the Declaration of Helsinki (as revised in Edinburgh 2000). Only remnant tissue was used for the project. All patients declare informed consent. The protocol was approved by the local ethics committee of the HUVR (CEI 2013/PI002). We report the availability of the ‘data set’ necessary to interpret, replicate and build on the findings reported in the paper. All of them are based on already public database conveniently described. All other reagents will be available to scientist upon request to validate these results.

### Cell lines and transfection

The T47D, HeLa, Calu3 and U937 cell lines were obtained from the European Collection of Animal Cell Cultures (ECACC) commercial repository at the beginning of the study (2012). No further authentication was performed by the authors. The CNIO AA and AW sarcoma cell lines have been generated in our lab and previously described [82]. Cell lines were transfected with pBABE to overexpress *MAP17* (T47D, HeLa, AA and AW) or shRNA targeting *MAP17* (Calu3) and were selected with 1 μg mL^-1^ of puromycin.

### Analysis of gene transcription

Total RNA was purified using the ReliaPrep^TM^ RNA Tissue Miniprep System (Promega, Fitchburg, WI, USA) according to the manufacturer's instructions. Reverse transcription was performed with 3 μg of mRNA using the High-Capacity cDNA Reverse Transcription Kit (Life Technologies) according to the manufacturer's recommendations. To detect changes in gene expression, we used the following probes, all from Life Technologies: *MAP17* (Hs00906696_m1), *HLA-A* (Hs01058806_g1), *HLA-B* (Hs00818803_g1), *HLA-C* (Hs00740298_g1), *BBS10* (Hs00379769_g1), *HERC2* (Hs00190589_m1), *ADNP* (Hs00209721_m1), *PYCARD* (Hs01547324_gH) and *GAPDH* (Hs03929097_g1). Quantitative PCR was performed and normalized as previously described [51]. At least three independent experiments were performed for each of the analyzed genes. Student's t-test was applied for each pair of samples, with a significance threshold of p < 0.05.

### Immunohistochemistry

IHCh analyses were previously described at [83–85]. The primary antibodies CD45 (NB110-93609, Novus Biologicals), CD68 (MCD497, Bio-Rad), CD4 (SP35, Roche) and CD8 (SP57, Roche) were incubated overnight at 4°C as previously described in [23, 33, 83]. Primary antibodies anti-MP17 was used at 1:4 dilution, as previously described [23]. A secondary antibody anti-goat (ab97100) for MAP17 was used. For CD45, CD68, CD4 and CD8 antibodies, the anti-rabbit (JI-111-035-003) was applied and revealed using substrate buffer and chromogen (Envision, Flex DAKO). The tissues were counterstained with hematoxylin (DAKO).

### Dendritic differentiation

Conditioned media from AW, Calu3 and AA cells were filtrated using a 0.45 μm pore size. 10^4^ U937 cells non-adherent cells were cultured for 72 hours in 6-well plates with 50% conditioned media, plus 50% RPMI. The plates were washed twice with PBS and fixed using glutaraldehyde 0.5%. After that, cells were stained with 1% crystal violet, washed twice with PBS and dried.

### Protein extraction and WB analysis

Western Blot analysis were obtained as described previously [51]. We used antibodies against IL-6 (Cell Signaling, #12912, 1:1000 dilution), NFAT2 (Cell Signaling, #8032, 1:1000 dilution) and α-Tubulin (Sigma, T9026, 1:10000 dilution). Horseradish peroxidase–labeled rabbit anti-mouse (Abcam, ab97046, 1:5000 dilution) and goat anti-rabbit (Abcam, ab97051, 1:5000 dilution) secondary antibodies were used.

### NFAT2 quantification

AW cells were cultured, fixed and permeabilized as previously described [50]. Then, anti-NFAT2 (Cell Signaling, #8032) were added to cells in 1 mL of PBS, 0.1% Triton X-100, 3% BSA at a 1:1000 dilution and incubated overnight at 4°C with gentle stirring. After that, coverslips were washed four times with PBS, 1% Triton X-100 for 5 minutes each time, with a final wash with PBS for 5 minutes. For NFAT2 quantification immunofluorescence assay, Alexa Fluor goat anti-rabbit IgG (A-11008, Life Technologies) was added at a 1:250 dilution to the cells in 1 mL of PBS, 0.1% Triton X-100, 3% BSA and incubated in dark at room temperature for 2 hours with gentle stirring. Cells were then washed three times (5 minutes each) with PBS, 0.1% Triton X-100. Finally, coverslips were mounted on a slide with a drop of mounting solution (Prolong Gold Antifade, Life Technologies) and dried. NFAT2 images were acquired in an Olympus BX61 fluorescence microscope. Nuclear NFAT2 was quantified using ImageJ software and expressed as the fluorescence in nuclei relative to the normalized cytoplasm fluorescence.

### Bioinformatics analysis

To find genes correlated with *MAP17*, we selected 5 databases for cervical cancer, 6 databases for lung cancer, 19 databases for breast cancer and 10 databases for colon cancer (see Supplementary Table 2). All of these databases are freely accessible through the R2 webpage (R2: Genomics Analysis and Visualization Platform (http://r2.amc.nl).

#### Correlations with MAP17

We looked for correlations with *MAP17* (*PDZK1IP1*, 219630_at), using an R-value lower than 0.05 in order to find significant differences. To find gene expression correlations, we used probe 219630_at, which corresponds to *MAP17*, for all Affymetrix datasets. For the TCGA and Budinska datasets, we used a unique probe for the *MAP17* gene.

From the list of correlated genes, we separated genes positively correlated with *MAP17* from genes negatively correlated with *MAP17*, generating two gene lists for each database. Next, we looked for genes highly represented among the different datasets. Thus, we established a cutoff for each gene of appearing at least in two different databases in cervical cancer, three different databases in lung cancer, and five different databases each in breast and colon cancer. We thereby generated four groups of genes commonly negatively or positively correlated with *MAP17*. To generate a Venn diagram to find common genes correlated with *MAP17* for all tumors, we used the tool Venny (*Oliveros, J.C. (2007-2015) Venny. An interactive tool for comparing lists with Venn's diagrams*. http://bioinfogp.cnb.csic.es/tools/venny/index.html).

#### Gene ontology (GO) analysis

To identify which pathways or Gene Ontology (GO) terms were connected to genes positively or negatively correlated with *MAP17*, we used enrichment analysis from the Gene Ontology consortium webpage (http://geneontology.org/page/go-enrichment-analysis). To find the subcellular locations of the proteins encoded by the genes identified as correlated with *MAP17*, we used the UniProt server (http://www.uniprot.org/).

The expression of individual genes in different databases was analyzed using MegaSampler software, included in the R2 server. For that purpose, we used the following probes: *MAP17* (219630_at), *HLA-A* (213932_x_at), *HLA-B* (204140_x_at), *HLA-C* (216526_x_at), *BBS10* (219487_at), *HERC2* (217902_s_at) and *ADNP* (226426_at). As controls for lung inflammatory diseases, we used the normal datasets Shelhamer (GSE39061) [86] and Salazar (GSE2125) [87], while datasets from Wang (Mixed Barrett's Esophagus, GSE26886) [88], Tiley (COPD, GSE43939) [89] and Meltzer (Lung Fibrosis, GSE24206) [90] were used to analyze the gene profile in disease status. To analyze the expression of the same set of six genes in colon inflammatory diseases, we selected Vivier dataset (GSE41469) [91] as a control, along with one database for Crohn's Disease (Arijs, GSE16879) [92] and three databases for Colon Inflammation (Seidelin, GSE9452 [93]; Sleiman, GSE10616 [94] and Salas, GSE38713 [95]) were used for disease state. In this case, we used a probe for *HLA-J* (217436_x_at), another gene positively correlated with *MAP17*. All data were normalized using the MAS5.0 algorithm and are shown as the log2 transform, performed automatically by R2 software.

#### R bioconductor analysis

To examine whether this inflammatory profile is connected to *MAP17* overexpression in other tumors, we selected another four cancer databases (GSE9574, breast cancer; GSE6008, ovarian cancer; GSE27155, thyroid cancer; GSE55945, prostate cancer). For this analysis, we did not consider non-tumoral samples. For the ovarian cancer dataset, we selected serous adenocarcinoma samples, while for thyroid cancer, we selected follicular variant thyroid gland papillary carcinoma samples. Each whole dataset was normalized using the RMA algorithm in Bioconductor, and tumoral samples were separated into two groups: low or high expression of *MAP17*. To analyze the expression of the genes identified above, we used the same probes, with the exception of *HLA-B* (208729_x_at) and *ADNP* (201773_at), because in at least one of the databases used, the previously used probe did not appear. In addition, as controls, we used *ACTB* (200801_x_at) and *TUBA1B* (211072_x_at). *PYCARD* (221666_s_at) and *CASP1* (206011_at), two components of the inflammasome platform, were also analyzed. To analyze the possible connection between *MAP17* and inflammatory diseases, we analyzed mRNA levels in three different databases: GSE52471 for psoriasis and discoid lupus [96], GSE13887 for lupus [97], and GSE20735 for a lupus model in mice [98]. To detect significant differences, Student's t-test was applied for each pair of samples, with a significance threshold of p < 0.05.

## SUPPLEMENTARY MATERIALS FIGURES AND TABLES


