# Supplementary material for: The cargo protein MAP17 (PDZK1IP1) regulates the immune microenvironment
Source: Oncotarget. 2017 Oct 6;8(58):98580–97. doi: 10.18632/oncotarget.21651 (PMC5716752; doi:10.18632/oncotarget.21651)
Supplement: Supplementary file 1 [file oncotarget-08-98580-s001.pdf]

The cargo protein MAP17 (PDZK1IP1) regulates the immune microenvironment

SUPPLEMENTARY MATERIALS

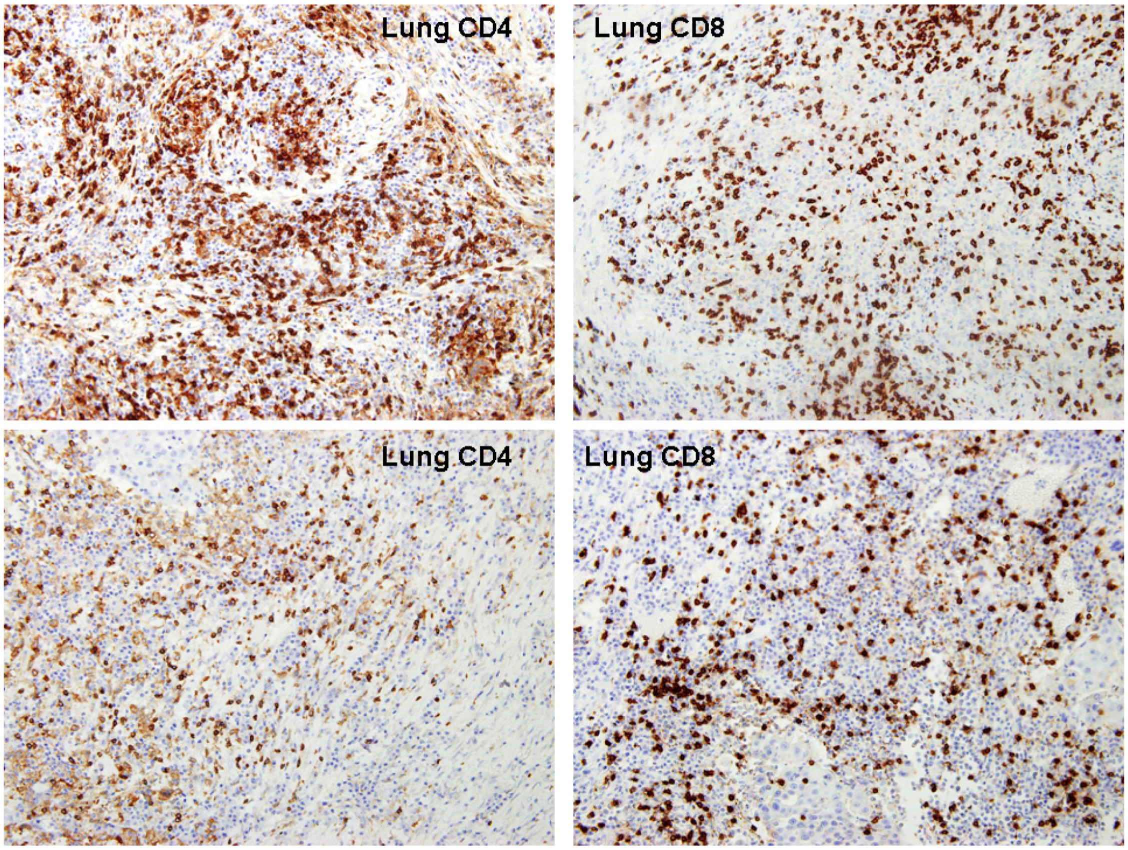

Supplementary Figure 1: Images of Lung tumor samples expressing CD4+ and CD8+ T cells. The primary antibodies CD4 (SP35, Roche) and CD8 (SP57, Roche) were incubated overnight at 4ºC as described in M&M. A secondary antibody anti-rabbit (JI-111-035-003) was applied and revealed using substrate buffer and chromogen (Envision, Flex DAKO). The tissues were counterstained with hematoxylin (DAKO).

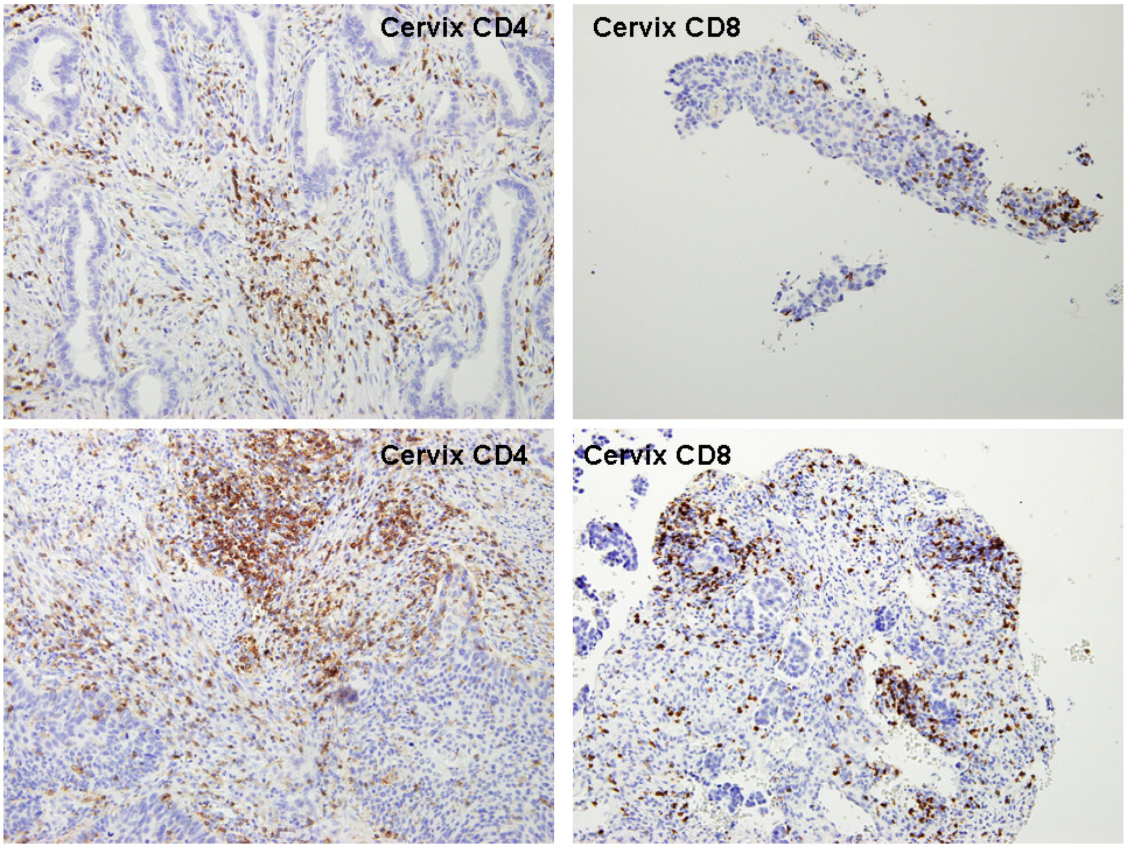

Supplementary Figure 2: Images of cervix tumor samples expressing CD4+ and CD8+ T cells. The primary antibodies CD4 (SP35, Roche) and CD8 (SP57, Roche) were incubated overnight at 4ºC as described in M&M. A secondary antibody anti-rabbit (JI-111-035-003) was applied and revealed using substrate buffer and chromogen (Envision, Flex DAKO). The tissues were counterstained with hematoxylin (DAKO).

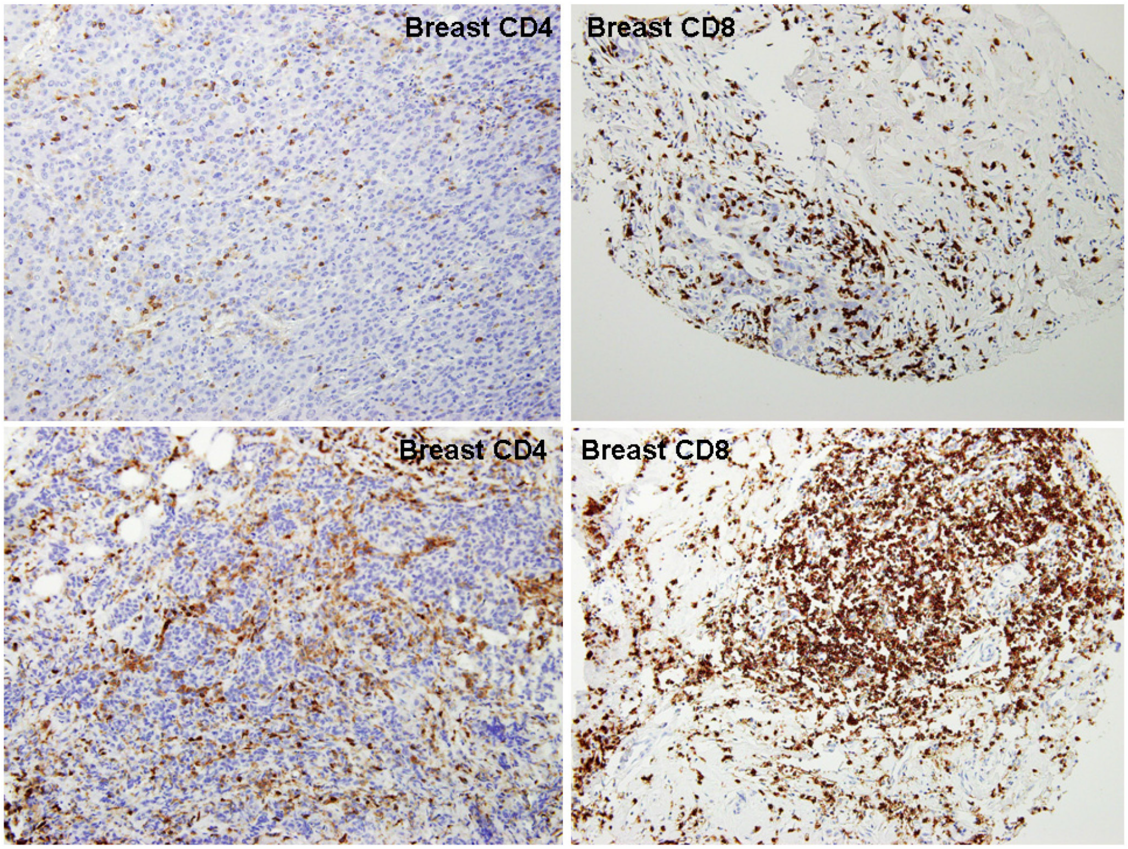

Supplementary Figure 3: Images of breast tumor samples expressing CD4+ and CD8+ T cells. The primary antibodies CD4 (SP35, Roche) and CD8 (SP57, Roche) were incubated overnight at 4ºC as described in M&M. A secondary antibody anti-rabbit (JI-111-035-003) was applied and revealed using substrate buffer and chromogen (Envision, Flex DAKO). The tissues were counterstained with hematoxylin (DAKO).

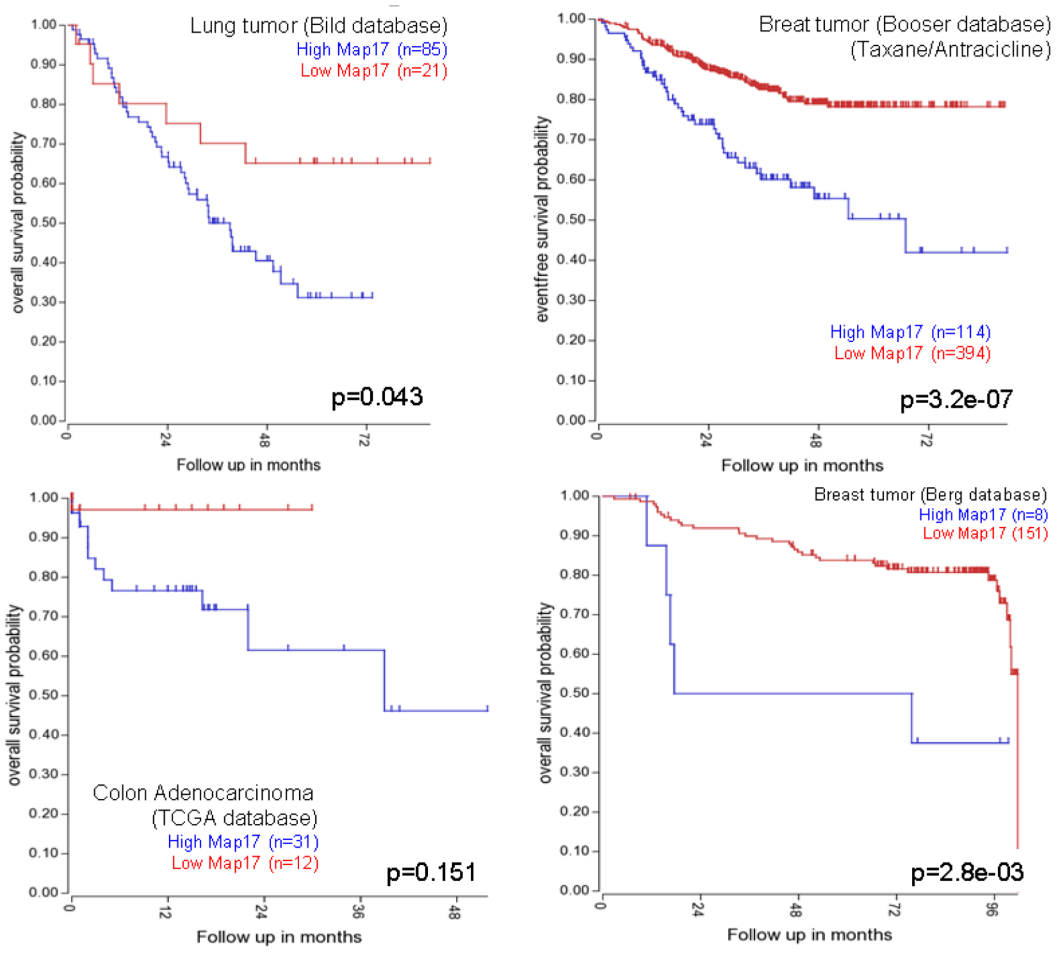

Supplementary Figure 4: Survival probability of patients with different types of cancer according to MAP17 levels in different cancer patient cohorts as indicated.

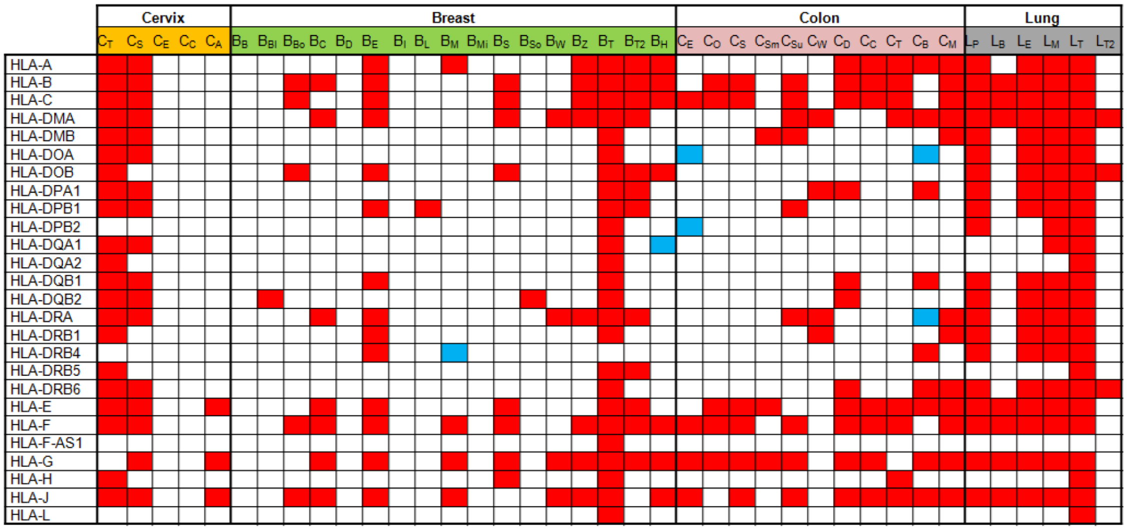

Supplementary Figure 5: *HLA* genes correlated positively (red) or negatively (blue) with MAP17 in all the datasets used in this study. C_T: TCGA 305; C_S: Schneider; C_E: EXPO; C_C: Cho; C_A: Ahlquist; B_B: Bergh; B_{Bl}: Black; B_{Bo}: Bos; B_C: Chin; B_D: Desmedt; B_E: EXPO; B_I: Iglehart; B_L: Loi; B_M: Miller 116; B_{Mi}: Minn; B_S: Servant; B_{So}: Sotiriou; B_W: Wang; B_Z: Zhang; B_T: TCGA 1097; B_{T2}: TCGA 528; B_H: Halfwerk; C_E: EXPO; C_O: Olschwang; C_S: Sieber; C_{Sm}: Smith; C_{Su}: Sugihara; C_W: Watanabe; C_D: Domany; C_C: Clary; C_T: TCGA; C_B: Budinska; C_M: Marisa; L_P: Peitsch; L_B: Bild; L_E: EXPO; L_M: Muley; L_T: TCGA 515; L_{T2}: TCGA 81. More than 90% of the correlations between MAP17 and a member of the HLA family are positive, suggesting that higher MAP17 levels may induce the expression of HLAs.

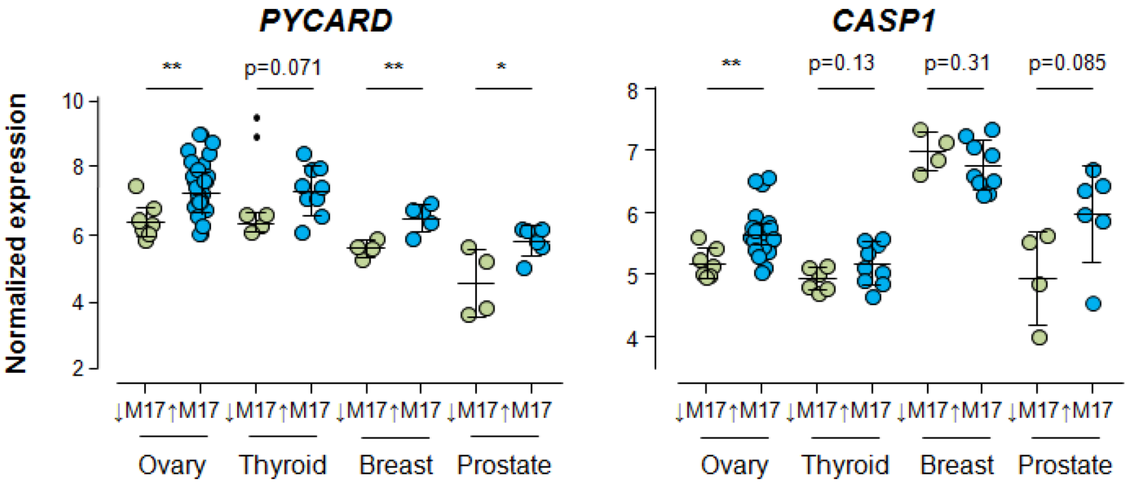

Supplementary Figure 6: mRNA expression levels of PYCARD and CASP1, two components of the inflammasome signaling platform, in breast, thyroid and ovarian cancer samples, grouped by differential expression levels of MAP17. Data were extracted from the bioinformatic analysis of several databases of the different tumors indicated. Although in some cases there are no significant differences, the tendency showed in most of the analysis.

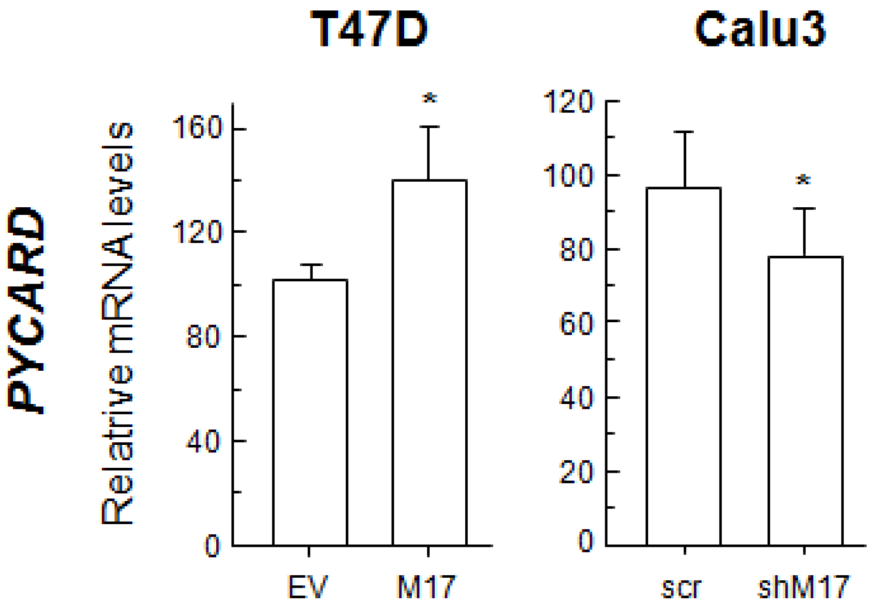

Supplementary Figure 7: *PYCARD* expression in cancer cells transfected for overexpression (T47D) or knockdown (Calu3) of MAP17. T47D breast cancer cell line was transfected with pBABE to overexpress *MAP17* while Calu3 lung cancer cell line (that endogenously expresses high levels of MAP17 was transfected with pRetroSuper carrying a shRNA targeting *MAP17* (Calu3). Both cell lines and were selected with 1 µg mL^{-1} of puromycin during 2 weeks and the mass culture subject to mRNA extraction. This mRNA was analyzed according to M&M procedures to analyze the levels of Pycard mRNA.

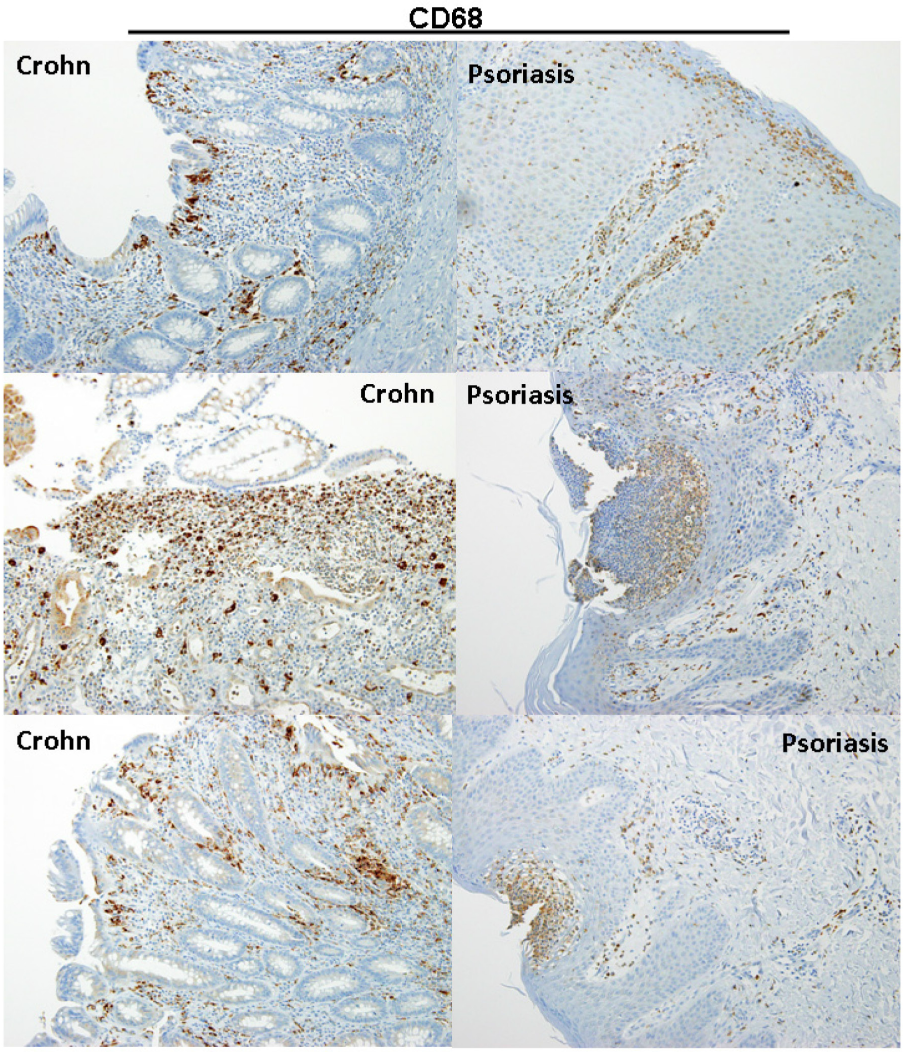

Supplementary Figure 8: Images of Crohn's and psoriasis samples expressing CD68 marker. The primary antibody against CD68 (MCD497, Bio-Rad), was incubated overnight at 4ºC as previously described in M&M. A secondary antibody anti-rabbit (JI-111-035-003) was applied and revealed using substrate buffer and chromogen (Envision, Flex DAKO). The tissues were counterstained with hematoxylin (DAKO).

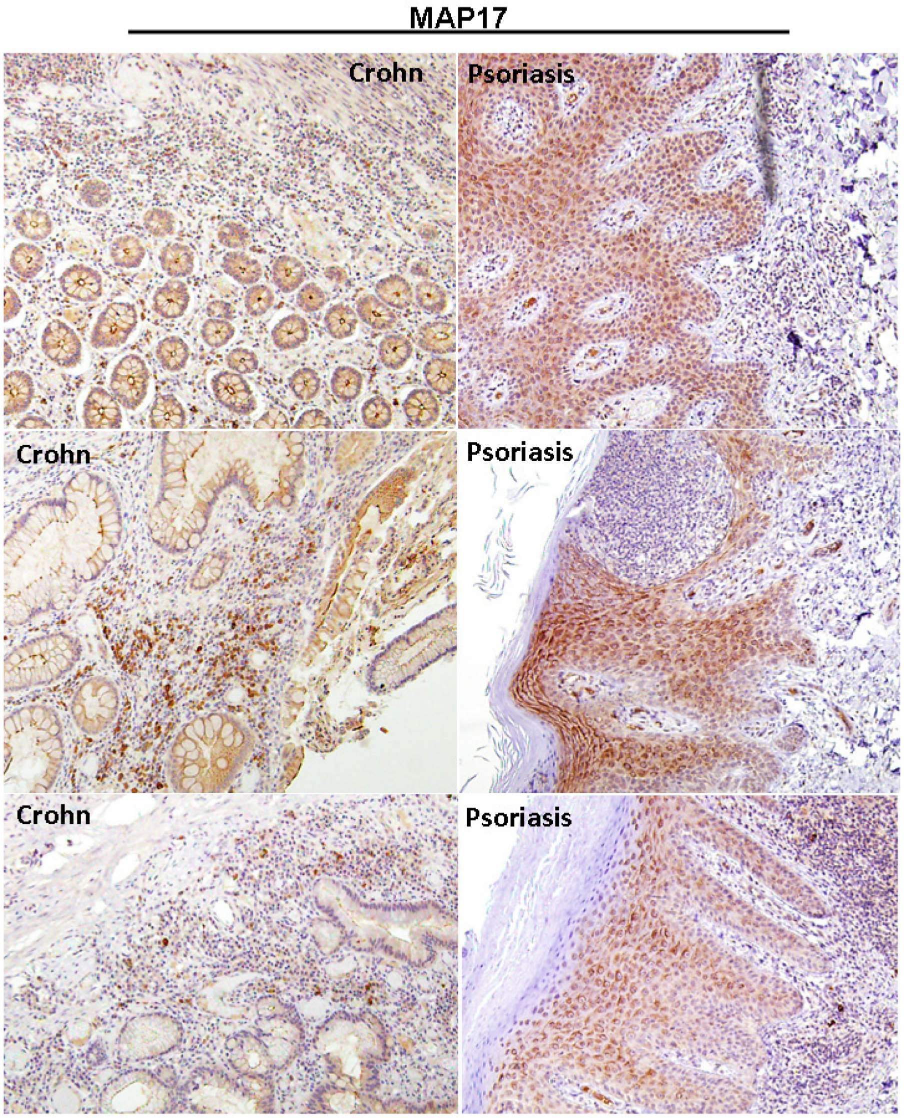

Supplementary Figure 9: Images of Crohn's and psoriasis samples expressing MAP17. The primary antibody anti-MP17 was used at 1:4 dilution, as previously described. A secondary antibody anti-goat (ab97100) for MAP17 was applied and revealed using substrate buffer and chromogen (Envision, Flex DAKO). The tissues were counterstained with hematoxylin (DAKO).

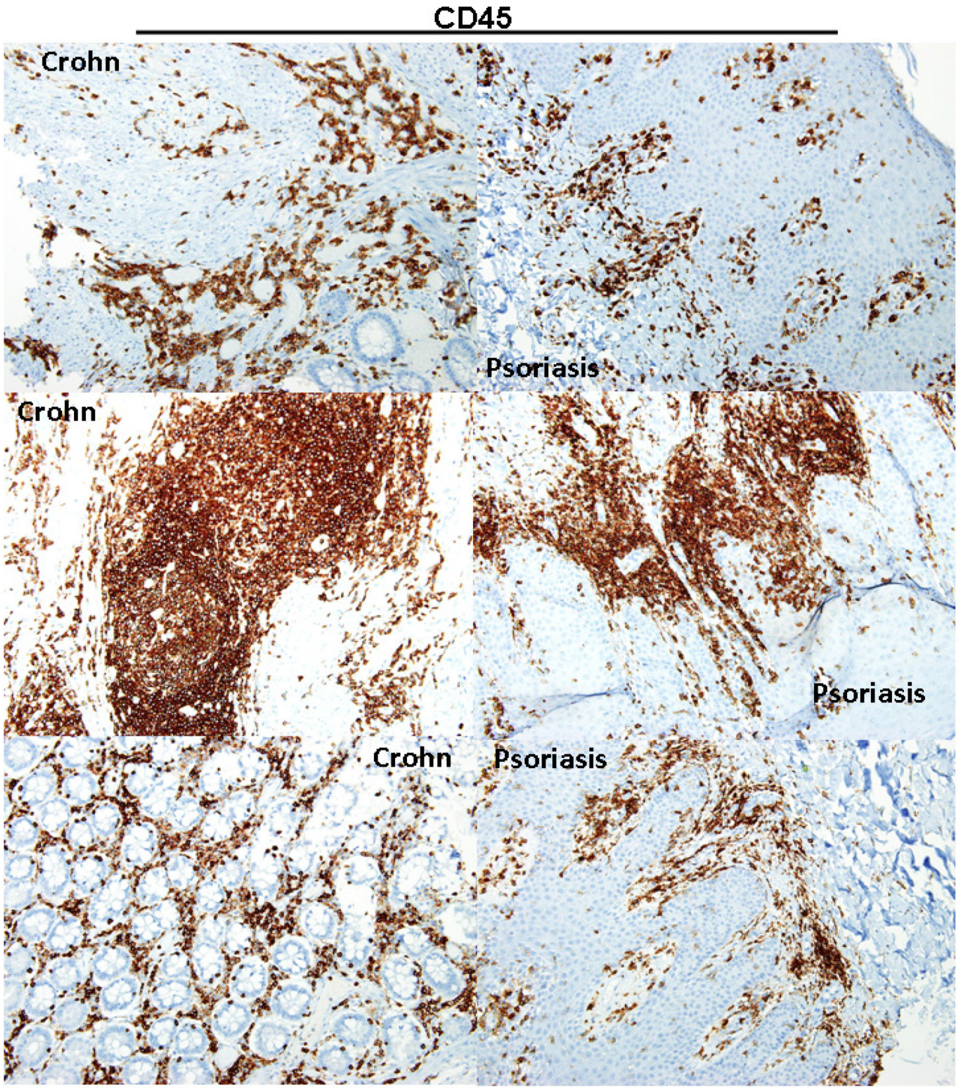

Supplementary Figure 10: Images of Crohn's and psoriasis samples expressing CD45. The primary antibody against CD45 (NB110-93609, Novus Biologicals), was incubated overnight at 4ºC as previously described in M&M. A secondary antibody anti-rabbit (JI-111-035-003) was applied and revealed using substrate buffer and chromogen (Envision, Flex DAKO). The tissues were counterstained with hematoxylin (DAKO).

Supplementary Table 1: MAP17 expression correlation to CD4, CD8 or the ratio CD4/CD8

MAP17 correlation to	Spearman			Pearson		
	CD4	CD8	CD4/CD8	CD4	CD8	CD4/CD8
Number of XY Pairs	53	52	55	53	52	55
Pearson r	0,04699	-0,08144	0,1298	0,07468	0,1091	0,1987
95% confidence interval	-0.2262 to 0.3134	-0.3467 to 0.1959	-0.1404 to 0.3820	-0.2076 to 0.3454	-0.1769 to 0.3781	-0.07838 to 0.4473
P value (two-tailed)	0,7383	0,5660	0,3449	0,5951	0,4413	0,1459
P value summary	ns	ns	ns	ns	ns	ns

MAP17 expression was quantified in two independent sections of each sample, according to the intensity of the signal (0= no expression, to 3= very high expression), by double blind observation of two independent pathologists. Equally, two independent sections of each sample were stained with antibodies against CD4 or CD8. CD4+ or CD8+ cell infiltration was valuated as percentage of total number of cells in the section. Then the correlation between MAP17 levels and percentage of infiltrating cells evaluated either assuming Gaussian distribution (Pearson test) or non-parametric correlation (Spearman test). Both data are shown.

Supplementary Table 2: List of datasets used in this work

Lung	Cervix	Breast	Colon
GSE43580 (Peitsch)	GSE6791 (Ahlquist)	GSE12276 (Bos)	GSE2109 (EXPO)
GSE3141 (Bild)	GSE2109 (EXPO)	GSE2109 (EXPO)	GSE37892 (Olschwang)
GSE2109 (EXPO)	GSE7803 (Cho)	GSE5460 (Iglehart)	GSE14333 (Sieber)
GSE33532 (Muley)	GSE9750 (Schneider)	GSE5462 (Miller)	GSE17538 (Smith)
TCGA/LUAD/515 samples	TCGA/CESC/305 samples	TCGA/BRCA/1097 samples	TCGA/COAD/286 samples
TCGA/LUSC/81 samples		GSE2034 (Wang)	GSE41568 (Clary)
		GSE30682 (Servant)	E-MTAB-990 (Budinska)
		TCGA/528 samples	GSE13294 (Jorissen)
		Halwerk (compendium)	GSE39582 (Marisa)
		GSE1456 (Bergh)	GSE41258 (Domany)
		GSE36771 (Black)	GSE21510 (Sugihara)
		CAARRAY (Chin)	
		GSE16391 (Desmedt)	
		GSE9195 (Loi)	
		GSE3494 (Miller)	
		GSE2603 (Minn)	
		GSE12093 (Zhang)	
		GSE7390 (Sotiriou)	

Supplementary Table 3: Genes correlated with MAP17 in breast, cervical, colon and lung tumors ($p < 0.05$)

Negative correlation				Positive correlation			
AAR2	*ACSL5*	*CASP8*	*CXCL1*	*FUT2*	*HLA-J*	*MPZL1*	*RIMS3*
ADNP	*AGPAT2*	*CCL20*	*CYBA*	*FUT3*	*HS3ST1*	*MTMR11*	*S100A10*
BBS10	*ALOX5*	*CD82*	*DAPP1*	*FUT6*	*HSD17B2*	*NCOA7*	*SAT1*
C18ORF54	*ANXA2*	*CDH3*	*EFNA1*	*GALE*	*IFNGR1*	*NFKB1*	*SELENBP1*
CBX1	*ANXA4*	*CEACAM1*	*EHF*	*GALNT3*	*IL10RB*	*NMI*	*SERPINA1*
CEP290	*APOL6*	*CEBPD*	*ELF3*	*GNA15*	*IL17RC*	***PDZK1IP1***	*SGMS1*
CHD6	*BCL10*	*CFB*	*EPHA1*	*GNG12*	*IL1B*	*PHLDA2*	*SH3BGRL3*
DDHD2	*BIRC3*	*CIB1*	*EPHA2*	*GUK1*	*IL32*	*PLEK2*	*SLC28A3*
HDGFRP3	*C1orf106*	*CLDN4*	*EPS8L2*	*HECTD3*	*LAMB3*	*PLSCR1*	*SLC37A1*
HERC2	*C1orf116*	*CLDN7*	*ERP44*	***HLA-A***	*LAMC2*	*PMM2*	*SLC44A3*
MBTD1	*CAP1*	*CLIC3*	*ETHE1*	***HLA-B***	*LCN2*	*PROM2*	*SLC6A14*
NAP1L1	*CAPG*	*CLTB*	*FAS*	***HLA-C***	*LGALS3*	*PRSS8*	*SLPI*
SERPINI1	*CAPN2*	*CMPK1*	*FCGBP*	***HLA-E***	*LSR*	*PSMB9*	*SPINT1*
SOGA1	*CARHSP1*	*CSTB*	*FHL2*	***HLA-F***	*LY75*	***PYCARD***	*SQRDL*
USP11	***CASP1***	*CTSS*	*FOSL2*	***HLA-G***	*MALL*	*RARRES3*	*SRD5A3*
ZCCHC3							
ZFHX3							

Supplementary Table 4: List of genes negatively correlated with MAP17 in at least three of the four tumors considered

Lung, breast and colon	Breast, colon and cervix	Lung, breast and cervix	Lung, colon and cervix	
ACVR2B	*ADCY1*	*ATP2B1*	*MLLT11*	*CCSAP*
BEX1	*CBX6*	*PHF21B*	*KBTBD6*	*ZFR*
BPTF	*COL2A1*	*CBFA2T2*	*C2orf44*	*AKT3*
CAPRIN2	*COPS8*	*PGAP1*	*FXR1*	*GLI2*
CEP250	*DDX42*	*HNRNPA0*	*FBXO30*	*TET1*
CTNNBL1	*DPY19L2P2*	*CCDC34*	*PFN2*	*FAM216A*
DDHD1	*ECM2*	*TMEM194A*	*FAM161A*	*EXTL2*
FEM1B	*EID1*	*IFT52*	*HSF2*	*MIB1*
FGFR1	*FAM13B*	*LATS1*	*KLHL15*	*TOP2B*
GNAZ	*FAM179B*	*TIMELESS*	*ERCC3*	*ZBTB39*
HOXD8	*GPR137C*	*N4BP2*	*PHF3*	*MAP3K7*
KRR1	*KCNMA1*	*HNRNPH3*	*FAM171B*	*ZNF711*
MARK4	*LUC7L*	*DSCC1*	*PHIP*	*MAP1B*
MYEF2	*LUC7L3*	*INTS2*	*ABI2*	*GPR161*
NCOA6	*MDM1*	*TERF1*	*MCM8*	*HLTF*
NFYB	*MPDZ*	*SART3*	*MSH6*	*LSM14A*
PCDH19	*MYH10*	*RAD51C*	*DNM1L*	*MYL6B*
PGBD1	*NBEA*	*TBL1X*	*PHKA2*	*COQ10A*
	NEK9	*NETO2*	*MAGEF1*	*KPNA5*
	PCNXL4	*BCL7A*	*LARP6*	*KIF5C*
	PKD2	*DHX40*	*WASF1*	*GNAS*
	PPP1R12A	*TRIM37*	*GPRC5B*	*USP13*
	RBM39	*KAT6B*	*ORC3*	*TSPYL2*
	SALL2	*TMEM97*	*DLX2*	*NBPF1*
	SENP7	*RBM12B*	*TUBB2B*	*DPY19L2*
	SLC35E3	*AP3M2*	*LONRF1*	*CSRNP2*
	SLC39A6	*MNAT1*	*HNRNPA3*	*LOC646762*
	SS18L1	*NFS1*	*AHCTF1*	
	TMEM55A	*GDF11*	*ZNF507*	
	TRPC1	*RALGAPB*	*ASAP1*	
	TSPYL4	*BRD3*	*APMAP*	
	TTC28	*MPP2*	*NUDT11*	
	TUBA1A	*PATZ1*	*ZNF594*	
	TULP4	*DHX30*	*SHPRH*	
	ULK2			
	ZBTB10			
	ZFP30			

Supplementary Table 5: List of genes positively correlated with MAP17 in at least three of the four tumors considered

Lung, breast and colon		Breast, colon and cervix		Lung, breast and cervix		Lung, colon and cervix		
ADAM15	*LYN*	*ABLIM1*	*MFSD6*	*ACE2*	*HSPA6*	*AGPAT3*	*GPRC5A*	*REEP5*
AKR1A1	*MOCOS*	*AK2*	*MOGAT2*	*ALDH1A3*	*IFI16*	*AGTRAP*	*GRN*	*RHOA*
ANG	*MSLN*	*AKR1B10*	*NDRG1*	*ALOX5AP*	*IFIH1*	*AIM1*	*HCP5*	*RNF19B*
ANXA3	*MST1R*	*ANXA2P2*	*NMRK1*	*ALS2CL*	*IL15*	*AK1*	*HYAL1*	*S100A14*
ARPC1B	*MUC1*	*ARHGAP26*	*OLFM4*	*AQP3*	*IL4R*	*ALDH2*	*IFI27*	*S100A16*
ASS1	*MUC20*	*ARHGEF5*	*OSTF1*	*ARHGDIB*	*KCNK5*	*ANXA11*	*IFI35*	*SCO2*
AZGP1	*MUC5B*	*ARPC1A*	*PDLIM1*	*BRI3*	*LY6D*	*AP1M2*	*IFITM1*	*SCP2*
B3GNT3	*NEK6*	*ARPC5*	*PFKFB2*	*BTN3A3*	*LYNX1*	*ARHGEF10L*	*IL18*	*SDR16C5*
BACE2	*PAPSS2*	*BSPRY*	*PFKP*	*C1R*	*MAP3K6*	*ARL14*	*IL1A*	*SERINC2*
CCL28	*PARP3*	*CBLC*	*PI3*	*CASP4*	*MLKL*	*ARPC3*	*IL1RN*	*SERPINB1*
CD55	*PCSK7*	*CBR3*	*PITX1*	*CD14*	*MPZL2*	*ARRDC1*	*IL2RG*	*SERPINB6*
CDC42EP1	*PDXK*	*CD58*	*PKP3*	*CD59*	*MSRA*	*ARSD*	*IL7*	*SIDT1*
CDCP1	*PEX11B*	*CTNNBIP1*	*PLEKHG6*	*CFI*	*MYD88*	*ASL*	*INPP1*	*SLCO4A1*
CREB3L1	*PIGR*	*DIAPH1*	*POU2F3*	*CFLAR*	*MYO1E*	*ATP10B*	*INPP4B*	*SSH3*
CXCL2	*PLAUR*	*DSC2*	*PPARA*	*CHI3L2*	*NFKBIE*	*ATP6V0D1*	*IRF1*	*STAT6*
DAP	*PLCH1*	*DUOX1*	*PRSS16*	*CLEC5A*	*NOD2*	*B3GALT4*	*IRF8*	*STX4*
DTX2	*PLXNB2*	*EGLN3*	*PRSS22*	*CLEC7A*	*NPAS2*	*BAK1*	*ISG20*	*SYNGR2*
DTX4	*PPA1*	*ERO1L*	*PSMB2*	*CNDP2*	*NUCB1*	*BLNK*	*ITGA3*	*TCN1*
EFHD2	*PPP1R1B*	*ETS2*	*PTPRF*	*CREG1*	*OPTN*	*C15orf48*	*KCNK6*	*TJP3*
FMO5	*PSMB10*	*F11R*	*PVRL4*	*CST6*	*OSMR*	*C19orf33*	*LGALS3BP*	*TLR3*
FRK	*PSMB8*	*F12*	*RAB25*	*CTSC*	*PHF11*	*C1orf210*	*LGALS9*	*TMEM159*
GGT6	*RAB20*	*FGFBP1*	*RASAL1*	*CTSD*	*PLIN3*	*C4BPA*	*LRG1*	*TMEM45B*
GLRX	*RNF145*	*GABRP*	*RHBDL2*	*CXCL16*	*RAB27A*	*CA2*	*LRP10*	*TMEM50A*
GOLM1	*RORC*	*GIPC1*	*RTP4*	*DEFB1*	*RAB38*	*CARD10*	*LXN*	*TMEM59*
GOLPH3L	*RPS6KA1*	*GJB3*	*S100A11*	*DUSP22*	*RHOG*	*CASP5*	*LYZ*	*TNFRSF14*
GPRIN2	*S100A6*	*GJB5*	*S100P*	*ENDOD1*	*RIN2*	*CAST*	*MAL2*	*TNFSF13*
GSTK1	*SBNO2*	*GNG5*	*SDC1*	*EVA1C*	*S100A8*	*CATSPERB*	*MGLL*	*TNIP1*
HDHD3	*SH3GLB1*	*GSTP1*	*SERPINB5*	*FPR1*	*S100A9*	*CD47*	*MMP28*	*TOR4A*
IFNGR2	*SIAE*	*HEBP2*	*SERPINB7*	*FXYD5*	*SCEL*	*CDS1*	*MUC4*	*TPK1*
KCNN4	*SLC22A18*	*HMOX2*	*SFN*	*GLUL*	*SERPINA3*	*CEACAM6*	*MVP*	*TSPO*
KCNQ1	*SLC22A18AS*	*IDH2*	*SH2D4A*	*HLA-DMA*	*STAT3*	*CHP1*	*NFKBIZ*	*TST*
KDELR2	*SLC35C1*	*IMPA2*	*SLC5A1*	*HLA-DRA*	*TACSTD2*	*CLIC1*	*NUDT22*	*TSTA3*
KDELR3	*SLC41A2*	*IRF6*	*ST14*			*CNKSR1*	*OBFC1*	*TUBA4A*
LIF	*ST6GALNAC4*	*ITGB4*	*STAP2*			*DENND2D*	*PCDH1*	*UBE2L6*
LIPH	*TAGLN2*	*KLF5*	*STK24*			*DHCR24*	*PIK3C2B*	*VAMP8*
LITAF	*TAPBP*	*KLK10*	*STYK1*			*DOK4*	*PLEKHM1*	*VDR*
LMAN2	*TCIRG1*	*LAD1*	*SYPL1*			*ELMO3*	*PRR13*	*VILL*
LRRC31		*LDHA*	*TAPBPL*			*EPS8L1*	*PSME1*	*VNN1*
						ERBB3	*PTK6*	*XDH*
						FAM129B	*PTPN22*	*YIPF1*
						FBXO6	*RAB27B*	*ZNF185*
						GALNT12	*RAC1*	
						GBP3	*RAC2*	
						GCNT3	*RBM47*	

Supplementary Table 6: List of genes related to secretory pathways (only genes appearing in the 4 tumors)

Secreted	Extracellular exosome	Cell membrane	Membrane	ER/Golgi
ANXA2	*C1ORF116/SARG*	*ANXA4*	*CD82*	*ACSL5*
CCL20	*GALE*	*CAPN2*	*CIB1*	*AGPAT2*
CEACAM1	*MTMR11*	*CDH3*	*CLIC3*	*ERP44*
CFB	*PMM2*	*CEACAM1*	*CLTB*	*FUT2*
CXCL1	*S100A10*	*CLDN4*	*DAPP1*	*FUT3*
EFNA1	*SH3BGRL3*	*CLDN7*	***HLA-A***	*FUT6*
FAS		*CYBA*	***HLA-B***	*GALNT3*
FCGBP		*EFNA1*	***HLA-C***	*HS3ST1*
IL1B		*EPHA1*	***HLA-E***	*MALL*
IL32		*EPHA2*	***HLA-F***	***PYCARD***
LAMB3		*FAS*	***HLA-G***	*SERPINA1*
LAMC2		*GNG12*	***HLA-J***	*SGMS1*
LCN2		*IFNGR1*	*HSD17B2*	*SLC28A3*
LGALS3		*IL17RC*	*IL10RB*	*SRD5A3*
PRSS8		*PLSCR1*	*LY75*	
SERPINA1		*PROM2*	*MALL*	
SLPI		*PRSS8*	*MPZL1*	
SPINT1		*SLC28A3*	***PDZK1IP1***	
			RARRES3	
			SAT1	
			SELENBP1	
			SLC37A1	
			SLC44A3	
			SLC6A14	

Supplementary Table 7: GO terms associated with genes negatively correlated with *MAP17* in at least three of the four types of tumors

GO biological process complete	Fold enrichment	p-value
chromosome organization	3.23	0.00199
anatomical structure development	1.72	0.00777
developmental process	1.64	0.0353

Complementary to Figure 2B.
